# GRG5/AES Interacts with T-Cell Factor 4 (TCF4) and Downregulates Wnt Signaling in Human Cells and Zebrafish Embryos

**DOI:** 10.1371/journal.pone.0067694

**Published:** 2013-07-01

**Authors:** Ângela M. Sousa Costa, Isabel Pereira-Castro, Elisabete Ricardo, Forrest Spencer, Shannon Fisher, Luís Teixeira da Costa

**Affiliations:** 1 IPATIMUP - Institute of Molecular Pathology and Immunology of the University of Porto, Porto, Portugal; 2 Faculty of Medicine of the University of Porto, Porto, Portugal; 3 Johns Hopkins University School of Medicine, Institute of Genetic Medicine, Baltimore, Maryland, United States of America; 4 University of Pennsylvania School of Medicine, Philadelphia, Pennsylvania, United States of America; 5 ICAAM - Institute of Agricultural and Environmental Mediterranean Sciences, University of Évora, Évora, Portugal; Laboratoire de Biologie du Développement de Villefranche-sur-Mer, France

## Abstract

Transcriptional control by TCF/LEF proteins is crucial in key developmental processes such as embryo polarity, tissue architecture and cell fate determination. TCFs associate with β-catenin to activate transcription in the presence of Wnt signaling, but in its absence act as repressors together with Groucho-family proteins (GRGs). TCF4 is critical in vertebrate intestinal epithelium, where TCF4-β-catenin complexes are necessary for the maintenance of a proliferative compartment, and their abnormal formation initiates tumorigenesis. However, the extent of TCF4-GRG complexes’ roles in development and the mechanisms by which they repress transcription are not completely understood. Here we characterize the interaction between TCF4 and GRG5/AES, a Groucho family member whose functional relationship with TCFs has been controversial. We map the core GRG interaction region in TCF4 to a 111-amino acid fragment and show that, in contrast to other GRGs, GRG5/AES-binding specifically depends on a 4-amino acid motif (LVPQ) present only in TCF3 and some TCF4 isoforms. We further demonstrate that GRG5/AES represses Wnt-mediated transcription both in human cells and zebrafish embryos. Importantly, we provide the first evidence of an inherent repressive function of GRG5/AES in dorsal-ventral patterning during early zebrafish embryogenesis. These results improve our understanding of TCF-GRG interactions, have significant implications for models of transcriptional repression by TCF-GRG complexes, and lay the groundwork for in depth direct assessment of the potential role of Groucho-family proteins in both normal and abnormal development.

## Introduction

**Figure 1:**
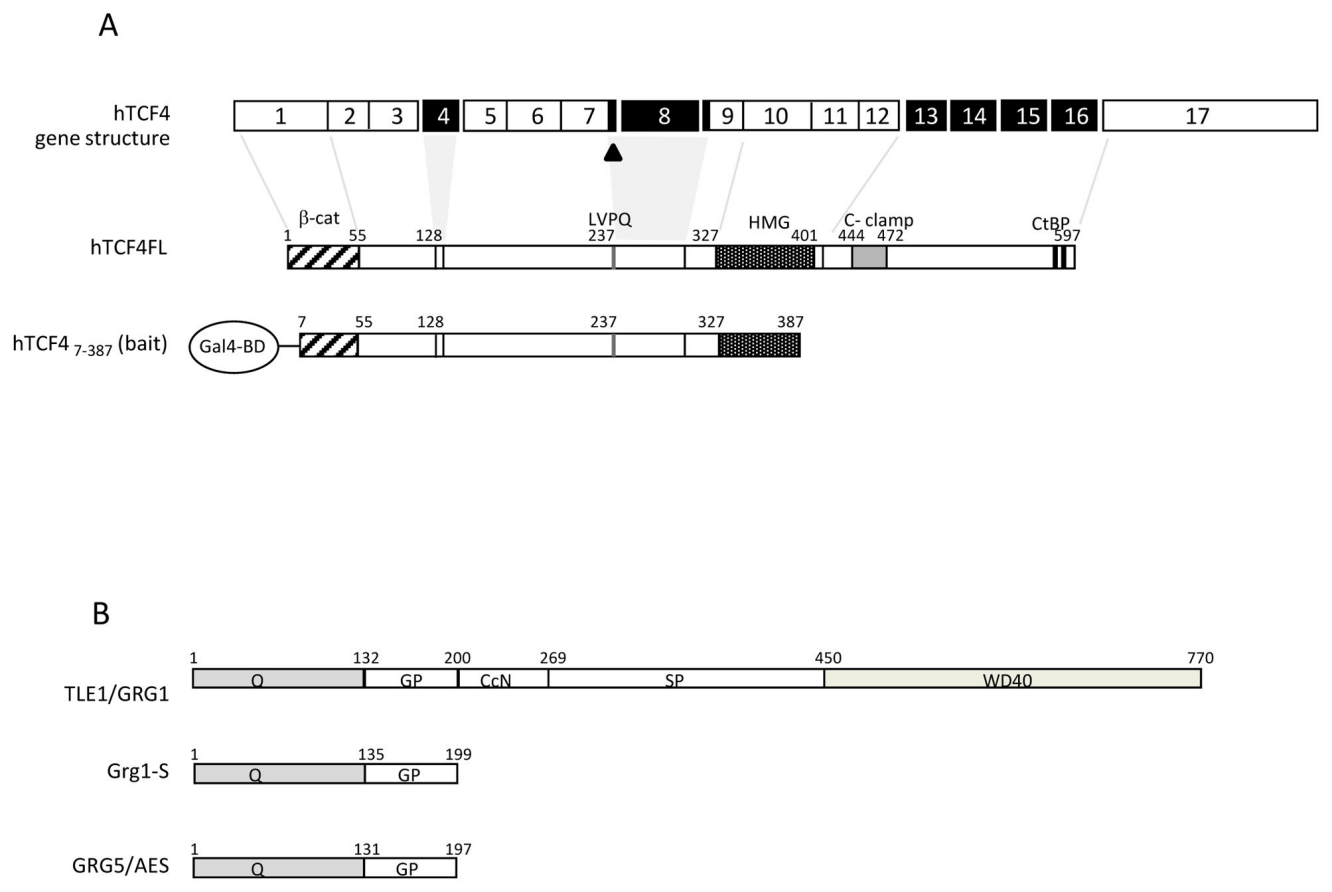
Schematic representation of the mammalian TCF4 and GRG domains organization. A) The human TCF4 gene consists of 17 exons (top), some of which are subject to alternative splicing (black exons). The TCF4 variant used (Ex1-17) includes isoform specific sequences such as the LVPQ domain due to the use of an alternative splice donor site at the end of exon 7 (arrowhead). A TCF4 fragment lacking the C terminus (a.a.s 7-387), was fused to Gal4’s DNA-binding domain (Gal4-BD) and used as bait in a two hybrid screen. B) The GRG family comprises two distinct classes of proteins, based on their domain constitution: the “long Grouchos”, GRG/TLE 1-4, consisting of five different domains (Q, GP, CcN, SP and WD40); and the “short Grouchos”, such as the alternative splice-product Grg1-S or the GRG5/AES subfamily members, which contain only the Q and GP domains.

The mammalian T-cell factor (TCF) family is composed of four members (TCF1, LEF1, TCF3 and TCF4 [1]), containing a highly conserved high-mobility-group (HMG) domain (Figure 1A), which is responsible for their ability to bind DNA specifically [2–4]. The first members of the family were cloned as regulators of T-cell receptor alpha enhancer in lymphocytes [5–7], but TCFs are now well recognized as important players in a wide variety of processes, especially in development [8–15]. TCF4 (encoded by the *TCF7L2* gene), in particular, is the most prominently expressed TCF/LEF member in the developing gut [11,16] and is necessary to maintain the proliferative compartment in the intestinal epithelium, as seen in TCF4-deficient mice and zebrafish [17–19].

A major breakthrough in our understanding of the TCFs’ roles and mechanism of action came with the discovery that they complex with β-catenin (encoded by the *CTNNB1* gene) to act directly as transcription factors, with the TCFs providing the DNA binding and β-catenin a potent transactivation domain [13,20,21]. This seminal discovery placed TCF-β-catenin complexes as the main effectors of Wnt signaling, a very important and evolutionary conserved pathway from *Drosophila* to humans [22–24]. Together with previous data on APC binding to and regulation of β-catenin [25–27], this led to the realization that abnormal activation of TCF-β-catenin-controlled transcription is the fundamental biochemical event underlying colorectal cancer initiation [18,28,29]. The summation of biochemical, developmental and oncobiology data thus led to a basic model of Wnt-dependent gene expression: upon Wnt signaling (Wg in *Drosophila* ), the integrity/assembly of a β-catenin phosphorylation complex containing APC, Axin/Conductin and GSK-3β kinase is affected [30–36]. This leads to a cytoplasmic accumulation of β-catenin and its translocation to the nucleus, where it interacts with TCF/LEF proteins to activate multiple target genes [11,15,30–40]. In the absence of Wnt, β-catenin phosphorylation targets it for degradation in the proteasome [41,42], freeing TCF proteins to participate in transcription repression [12,43–46].

The involvement of Groucho-family proteins in this repression was revealed by the findings that they interact with TCFs and antagonize TCF-mediated transcription activation and Wnt signaling in mammalian cells, as well as *Drosophila* and *Xenopus* embryos [47–49]. Importantly, these studies implied a role for GRGs in Wnt-mediated dorsal-ventral (DV) patterning [50,51], one of the major early developmental decisions made in vertebrate embryos, that requires β-catenin accumulation and signaling activation [43,52–58].

In vertebrates, the Groucho family can be divided into two distinct structural subgroups. The first includes the “long” proteins, termed GRG (1–4), for *Groucho-related gene* (or TLE (1–4), for *Transducin-like enhancer of split*, in humans), which have five distinct domains (Figure 1B): the highly conserved Q (glutamine-rich) and WD (containing WD40 repeats, and involved in most protein interactions) in the N- and C-termini respectively, separated by the much more variable GP (glycine/proline-rich), CcN (with putative casein Kinase II/cdc2 phosphorylation sites and a nuclear localization signal), and SP (serine/proline-rich) domains. They are generally accepted as co-repressors of multiple transcription factors with crucial roles in diverse processes, such as segmentation, sex determination, embryo patterning and organogenesis [47,59–61]. The second subgroup encompasses the proteins sometimes referred to as the “short Grouchos”, including alternative splicing variants of the long forms, such as Grg1-S (a variant of mouse Grg1) and distinct members expressed from their own loci, such as GRG5 (also referred to as AES, for *Amino-terminal enhancer of* split). Proteins in this subgroup contain only the first two conserved domains (Q and GP) (Figure 1B) and have sometimes been suggested to act as dominant negatives of the long forms [49,62]. However, this view has been contradicted by the demonstration of transcriptional repression by several short forms, including Grg1-S, AES197 (a “truncated” sea-urchin Groucho homologue) and GRG5/AES [63–69].

Here, we characterize the TCF4-GRG5/AES molecular interaction, map the minimal interacting region in TCF4 to a 111-amino acid stretch and show that, in contrast to other Grouchos, GRG5/AES-binding depends on the 4-amino acid motif LVPQ. Interestingly, both this motif and the 111-amino acid core binding region are present only in some TCF4 isoforms. We further demonstrate that GRG5/AES acts as an efficient repressor of TCF-β-catenin signaling both in human cells and zebrafish embryos, capable of counteracting the effects of activated β-catenin both in axis duplication and DV patterning during zebrafish embryogenesis. These results broaden our understanding of the physical and functional interactions between TCFs and Groucho-family proteins, will help develop more accurate models of Wnt-signaling regulation by the latter, and pave the way for a detailed *in vivo* analysis of the role played by TCF-GRG complexes in intestinal development, homeostasis and tumorigenesis.

## Materials and Methods

### Plasmids

TCF4 bait plasmids were generated by PCR amplification of cDNA fragments coding for amino acids 7-387 (“TCF4”) or 33-387 (“dnTCF4”) of human TCF4 isoform 1 (NP_001139746.1 NCBI accession number) and cloning into pAS2 and pAS2-1, respectively (Clontech). pGAD10-βcat (containing the entire coding region of β-catenin) was recovered in a pilot screen for TCF4-interacting proteins using a commercial human fibroblast cDNA library in pGAD10 (Clontech). TCF4 fragments used in interaction mapping (e.g. TCF4_1-129_), as well as a human TCF1 cDNA fragment coding for amino acids 176-359, were PCR amplified and inserted in frame with the Gal4-binding domain (G4BD) in pAS3, a pAS2 derivative containing a single EcoRI restriction site. The ACT-GRG4-QGP (GRG4_1-221_) and GAD-Grg1S plasmids were generated by subcloning the relevant cDNA fragments into pACT2 or pGADT7 (Clontech), in frame with the Gal4-activation domain (G4AD).

Myc-tagged TCF4 and HA-tagged GRG5/AES mammalian expression plasmids were generated by subcloning of partial and full-length human cDNAs into CMV-promoter driven plasmids (Clontech), in frame with the respective epitopes. CMV-βcat (WT) was generated by removing β-catenin cDNA from pGAD10-βcat and subcloning into pCMV. CMV-βcatT41A (coding for a mutant β-catenin found in human tumors) was derived from CMV-βcat by site directed mutagenesis. Expression vectors for Grg1-S and Grg1-L [63] were obtained from Addgene (Addgene plasmids 11065 and 11067, respectively). CMV-G4VP was generated by subcloning the Gal4-VP16 fusion [70] into a CMV-promoter driven mammalian expression vector; G4BD.GRG5 and G4BD.Grg1-L were generated by removing the VP16 coding sequences from CMV-G4VP (yielding CMV-G4BD) and replacing them with those of GRG5/AES and Grg1-L.

TCF-Luc (containing three TCF consensus binding sites - TREs) and TCF*-Luc (with three mutant TCF binding sites) were previously described [71]. For pB15UT-Luc, 3 copies of the 5 Gal4 upstream activation sequences (UAS_G_) present in UAS_G_-βgal [70] were concatamerized and used to replace the TREs in TCF-Luc. For 15UTSV-Luc, the SV40 promoter from "trigger P5" [70] was PCR amplified and cloned into pB15UT-Luc between the UAS_G_ and the luciferase gene.

Plasmid templates to synthesize mRNAs for zebrafish embryo injections were generated by cloning of full length cDNAs for *TCF4*, *GRG5/AES* and an activated β-catenin (*βcatT41A*), as well as TCF4_33-596_ (“*FLdnTCF4*”) into pCS2plus.

All plasmids were sequenced to confirm that they had been accurately constructed. More detailed information about the constructs is available upon request.

### Yeast two-hybrid assays

A commercial human fetal brain cDNA library in pACT2 (Clontech), was screened by PEG/LiAcetate co-transformation [72] with the bait plasmid pAS2-TCF4 into the AH109 yeast reporter strain (which contains integrated copies of *ADE2* and *HIS3* reporter genes under control of Gal4-dependent promoters). Primary candidates were selected for their ability to grow in SC-Leu-Trp-Ade medium (BIO101). A secondary screen was performed by selection on medium also lacking histidine and containing 30mM of the HIS3 inhibitor 3-Amino-1,2,4-triazole (3-AT), (Sigma). False positives were screened against by co-transformation of individual candidate prey plasmids with the control bait plasmid pLAM, which codes for a G4BD-laminin fusion protein.

Mapping of the GRG5/AES -binding region in TCF4 was done by individually co-transforming each of the various TCF4-fragment encoding bait plasmids with a pACT2-GRG5 plasmid recovered in the library screen and selection on SC-Leu-Trp-Ade medium. For each fragment, the strength of the interaction was evaluated by spotting 60 individual colonies on media also lacking histidine and containing increasing concentrations of 3-AT. Evaluation of the effects of removing the LVPQ motif was done by co-transforming TCF4 bait constructs with either GRG4-QGP or Grg1-S encoding prey plasmids and spotting a mixture of 20 transformant colonies from each interaction tested on SC-Leu-Trp-Ade selective medium.

### Prey-plasmid recovery

Putative candidates selected in the secondary screen were grown in SC-Leu-Trp and plasmid DNAs extracted by glass bead lysis and ethanol precipitation. DNA from each yeast clone (containing a mixture of the bait plasmid and one or more prey plasmids) was transformed into *E. coli* cells by electroporation and colonies containing different prey plasmids identified by PCR/agarose gel electrophoresis screening with bait plasmid and prey plasmid specific primers. Plasmid DNA was isolated by a standard alkaline lysis method and used to identify inserts by sequencing.

### Cell line, transfections and luciferase assays

Human embryonic kidney (HEK) 293 cell line (ATCC number: CRL-1573) was maintained in Dulbecco’s modified Eagle’s medium (DMEM) (Invitrogen) supplemented with 10% FBS (fetal bovine serum) (Invitrogen) and 1% of a solution containing 10000 U/ml penicillin and 10000 µg/ml streptomycin (Invitrogen).

Transient transfections were performed using Lipofectamine 2000 reagent (Invitrogen). Cells were seeded at 1×10^5^ cells/ml into 24-well plates one day prior to transfection. The total amount of transfected DNA was adjusted in each case to 1.5 µg per well by addition of the appropriate backbone vector. Effector plasmids included: pCMV-GRG5/AES (0.4, 0.8 or 1.2 µg), pCMV-β-catenin (0.1 µg), pCDNA3.1-dnTCF4 (0.4 or 1.2 µg), Grg1-S (1.2 µg), Grg1-L (0.4 or 1.2 µg). ), CMV-G4BD (1.2 µg), CMV-G4BD.GRG5 (1.2 µg) and CMV-G4BD.Grg1-L (1.2 µg). Luciferase reporter plasmids included TRE-Luc (0.1 µg), TRE*-Luc (0.1 µg), pB15UT-Luc (0.1 µg) and 15UTSV-Luc (0.2 µg). In each case, a CMV-β-galactosidase plasmid (0.1 µg) was co-transfected to normalize for transfection efficiency. For each transfection, the total DNA was incubated with 3 μl of lipofectamine for 20 minutes and gently added to the cells. Twelve hours after transfection, the medium was replaced and 24 hours later cells were washed in PBS buffer and collected to determine luciferase and beta-galactosidase activities. Cell lysates were prepared using 100 μl of 1XRBL (Promega) per well and luciferase activity was assayed according to the manufacturer’s instructions (Promega). Beta-galactosidase assays [70] were performed in 96-well plates using 5 μl of each cell lysate, 45 μl of reaction buffer and 10 μl of ONPG (o-nitrophenyl-β-D-galactopyranoside, Sigma) solution and stopped by addition of 25 μl of 1M Na _2_CO_3_ (Sigma). After subtraction of the background, the luciferase activities were normalized against beta-galactosidase activities. Each transfection was performed independently three times and all the assays were done in triplicate.

### Co-immunoprecipitation

Whole-cell extracts were prepared from HEK293 cells co-transfected with 1 μg of pCMV-Myc-TCF4 and 1 μg of pCMV-HA-GRG5/AES expression plasmids. 500 µg of protein were incubated at 4 °C, for 2h and with rotation with 2.5 µg of mouse monoclonal anti-Myc antibody (Clontech). Immunoprecipitates were incubated for 30 min with Protein G-Sepharose beads 4 Fast Flow (GE Healthcare). After the incubation, the beads were washed three times with 1 ml of lysis buffer (140 mM NaCl, 2.7 mM KCl, 10 mM Na_2_HPO_4_, 1.8 mM KH_2_PO_4_, pH 7.4, 1% Triton X-100, 1% Nonidet P-40, 1 mM phenylmethylsulfonyl fluoride, 20 mM NaF, 3 mM sodium vanadate, 10 µg/ml aprotinin and 10 µg/ml leupeptin) and boiled in sample buffer (4% SDS, 20% glycerol, 3.5% 2-mercaptoethanol, 0.004% bromophenol blue and 0.125 M Tris HCl, pH 6.8).

For total protein lysates, 20 µg of protein were loaded per case. Proteins were separated by 12% SDS-PAGE, transferred onto a Hybond nitrocellulose membrane (Amersham Biosciences) and subjected to Western blot analysis. Briefly, membranes were blocked for 30 min with 5% nonfat milk in PBS + 0.5% Tween-20 and incubated for 2 h with rabbit polyclonal anti-HA or mouse monoclonal anti-Myc antibodies (Clontech) diluted 1/1000. HA epitope-tagged GRG5/AES and Myc epitope-tagged TCF4 were detected using horseradish peroxidase-conjugated secondary antibodies (Santa Cruz Biotechnology), diluted 1/2000, followed by ECL detection (Amersham Biosciences).

### Ethics statement

All experiments were conducted according to an animal protocol approved by the University of Pennsylvania Institutional Animal Care and Use Committee (#802394). Veterinary care was under the supervision of the University Laboratory Animal Resources of the University of Pennsylvania.

### Fish and mRNA microinjections

Wild-type fish and embryos were maintained essentially as described previously [73].

Plasmids were linearized and transcribed in vitro using the SP6 Message mMachine Kit (Ambion). Synthesized mRNAs were purified by phenol: chloroform extraction and isopropanol precipitation and injected into one-cell stage embryos, except for the β-catenin-induced double axis experiments, in which mRNAs were injected into one blastomere of eight-cell stage embryos. Embryos were injected according to a previously described procedure [74].

### Whole-mount in situ hybridization

WT and injected zebrafish embryos were collected at 70% of epiboly stage and fixed in 4% paraformaldehyde. The following antisense RNA zebrafish probes were used: *chordin* [75], *gata2* [76] and *sp5l* [77]. Whole-mount in situ hybridizations were carried out as described previously [78].

## Results

### GRG5/AES specifically interacts with TCF4

TCF4 is considered one of the most important regulatory genes in intestinal development and homeostasis in vertebrates. As part of our general interest in a detailed understanding of the functions played by TCF4 in these processes, we tried to identify novel TCF4-interacting proteins. Because we were particularly interested in potential differences among the sets of proteins that interact with TCF4 and those that bind its homologues, we decided to perform a two-hybrid screen similar to the one that led to the identification of β-catenin as a TCF1 partner [13]. A truncated form of TCF4 (7-387 amino acids) fused to the Gal4-DNA-binding domain (G4BD) was therefore used as bait to screen a Gal4-activation domain (G4AD)-fused human fetal brain cDNA library (Figure 1A). This TCF4 fragment retains the N-terminal β-catenin interacting domain, as well as most of the HMG-box, but lacks the C-clamp motif found in E-tail containing isoforms [79], as well as the C-terminal CtBP-binding domain [80]. Additionally, it includes alternative exons in the intermediate region and isoform-specific sequences such as the LVPQ motif, as a result of the use of an alternative splice donor site at the end of exon 7 [81,82] (Figure 1A).

**Figure 2:**
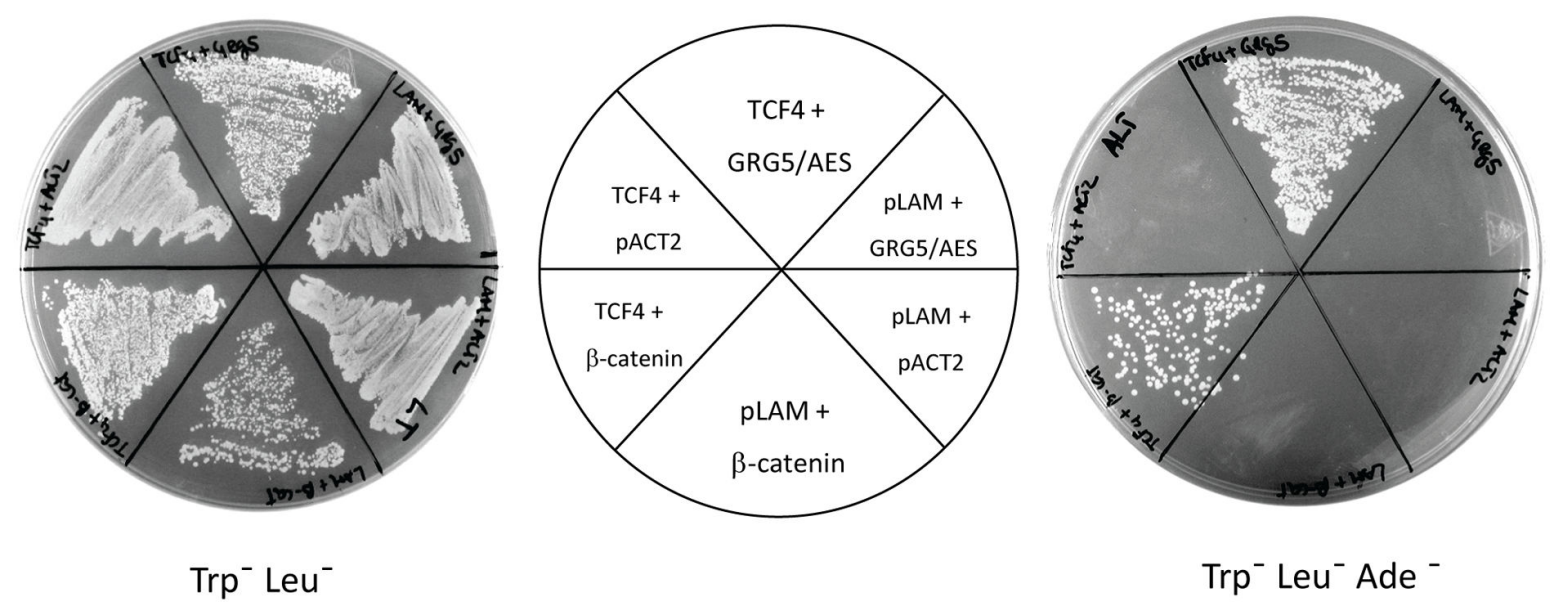
Physical interaction of TCF4 and GRG5/AES in yeast cells. The AH109 yeast strain was co-transformed with the indicated constructs (central panel). Transformants were selected in medium lacking tryptophan and leucine (left panel) and activation of a Gal4-dependent *ADE2* gene was examined by growth on selective plates additionally lacking adenine (right panel). Yeast cells containing both TCF4 and GRG5/AES grew in the selective media. pLAM and pACT2 plasmids were used as bait and prey negative controls respectively, while the β-catenin prey plasmid constitutes a positive control for the interaction assays.

Out of approximately 1.2 million independent yeast transformants initially screened, we isolated close to 300 candidate prey plasmids that were sequenced and analyzed by database comparison. The most frequently obtained cDNA, representing approximately 13% of the total and including many full-length clones, corresponded to GRG5/AES. This was similar to the results obtained with TCF1, but very surprising, in light of previous claims that mouse Grg5 binds TCF1 but not TCF4 [49]. To demonstrate that the TCF4-GRG5/AES interaction is specific, the TCF4 bait plasmid (pAS2-TCF4) was reintroduced into yeast cells along with pACT2 (prey “empty vector" expressing the G4AD). Conversely, the GRG5/AES prey plasmid (pACT2-GRG5) was reintroduced with pLAM control bait (encoding a fusion of laminin protein with G4BD). Figure 2 shows that yeast cells are only able to grow in the selective medium when co-transformed with both TCF4 and GRG5/AES fusion plasmids, indicating that a functional two-hybrid transcription factor is produced and therefore that TCF4 interacts with GRG5/AES.

**Figure 3:**
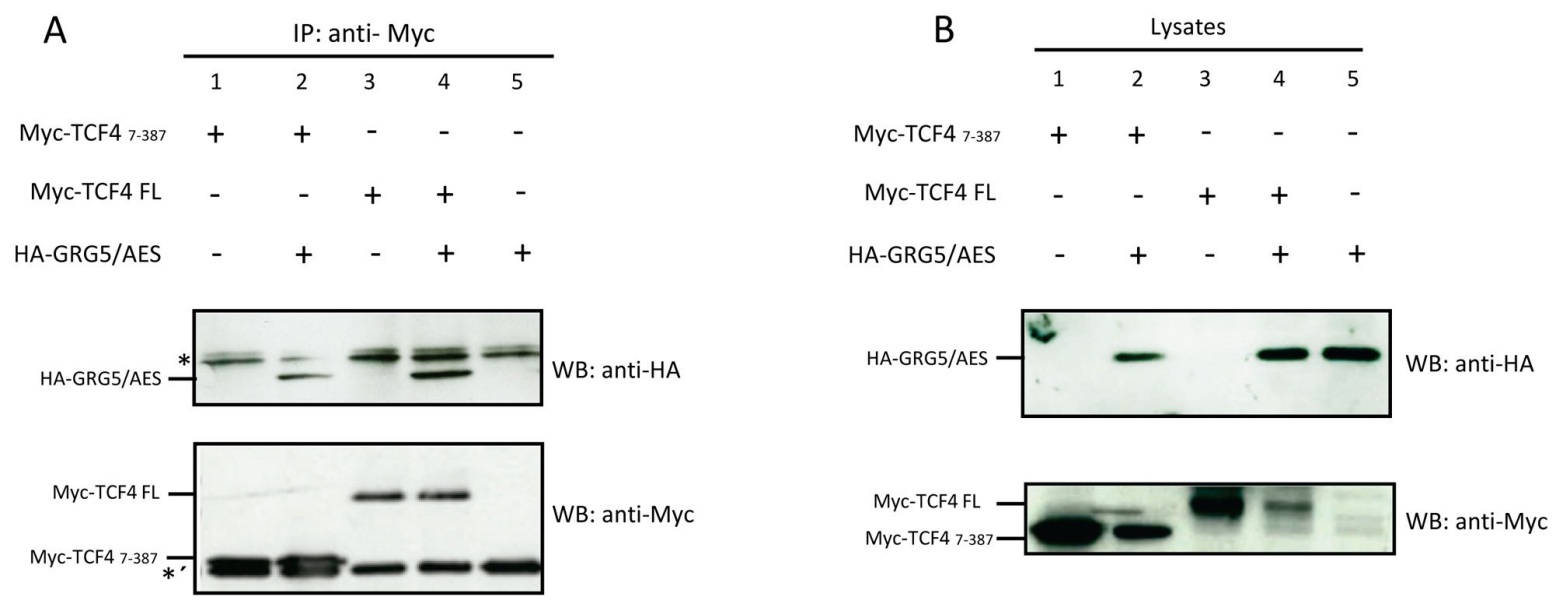
Physical interaction of TCF4 and GRG5/AES in human cells. A) Lysates of HEK293 cells transiently transfected with the indicated expression plasmids were subject to immunoprecipitation (IP) with anti-Myc antibody and the immunoprecipitated proteins were analyzed by Western blot (WB) with anti-HA and anti-Myc antibodies. Co-precipitation of HA-tagged GRG5/AES was detected only in the lysates of cells co-transfected with Myc-TCF4_7-387_ (lane 2) or Myc-TCF4FL (full-length) (lane 4). B) 10% of each total cell lysate was loaded for WB with either anti-HA or anti-Myc antibodies. The asterisks indicate nonspecific binding to immunoglobulin light (*) and heavy (*´) chains.

To determine if TCF4 and GRG5/AES can also interact in mammalian cells, co-immunoprecipitation studies were carried out on protein extracts of transiently transfected HEK293 cells (Figure 3). We expressed Myc-tagged TCF4 proteins either in the presence or absence of HA-epitope-tagged GRG5/AES. Full length TCF4 or the fragment containing residues 7-387 (used in the original two-hybrid screen), were precipitated from the cell lysates with a monoclonal anti-Myc antibody, and the immunoprecipitates were analyzed for the presence of both GRG5/AES and TCF4 proteins by Western blotting. GRG5/AES was readily and specifically detected in immunoprecipitates from co-expressing cells, as shown by the presence of the predicted 23kDa size protein (Figure 3A, lanes 2 and 4), but not from cells expressing only one of the proteins, confirming that there is a physical interaction between GRG5/AES and both full-length and truncated TCF4.

### TCF4_130-240_ is sufficient for strong interaction with GRG5/AES and other Grouchos

**Figure 4:**
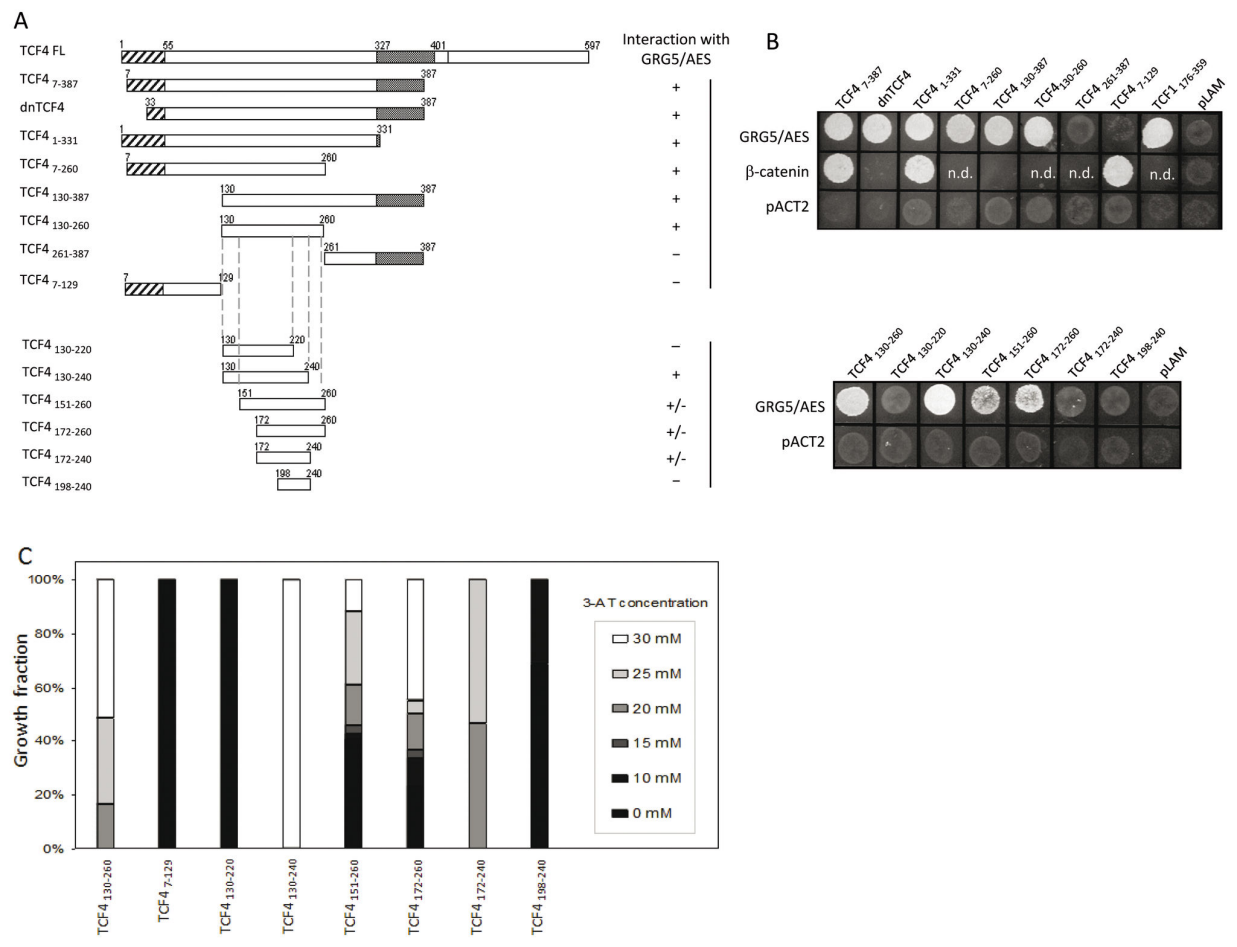
Mapping of TCF4’s GRG5/AES-interacting region using yeast two hybrid assays. A) Schematic diagram of the Gal4BD-TCF4 constructs tested for interaction with GRG5/AES. B) GRG5/AES-interacting TCF4 fragments activate an *ADE2* reporter gene, conferring to yeast transformants the ability to grow in selective medium lacking tryptophan, leucine and adenine. C) Transformants were also tested for activation of a *HIS3* reporter, as determined by growth in medium lacking histidine and with increasing doses of 3-aminotriazol (3AT). As defined by both reporter assays and summarized on the right panel of the diagrams, the region encompassing residues 130-240 is sufficient for the interaction, with residues 220-240 being strictly necessary. Two previously described fragments were included as internal controls: dnTCF4, that lacks the N terminal β-catenin binding domain [13]; and TCF1_176-359_ [49]; n.d.: not done.

To map the GRG5/AES-interaction domain of TCF4, we next fused successive truncations of TCF4 to G4BD (Figure 4A) and analyzed their ability to bind GRG5/AES by yeast two-hybrid (Figure 4). We first divided the original TCF4 bait fragment into two overlapping regions, TCF4_7-260_ and TCF4_130-387_ (Figure 4A), both of which were found to mediate the interaction (Figure 4B, lanes 4 and 5). We next tested the overlapping fragment, TCF4_130-260_, and found that this region is sufficient to mediate specific interaction with GRG5/AES (Figure 4B, lane 6). It was also shown to be necessary, as no interaction was detected with the flanking regions: TCF4_261-387_ failed to activate the reporter gene, as did TCF4_7-129_, despite its ability to interact with β-catenin (Figure 4B, lanes 7 and 8).

To gain further insight into this interaction, we next attempted to determine the minimal interacting region. Additional TCF4 truncations were thus generated in fusion with G4BD (Figure 4A) and tested by two-hybrid for adenine reporter activation (Figure 4B). Because the yeast strain AH109 is *HIS*- and contains an integrated copy of the *HIS3* gene under the control of a Gal4-dependent promoter, we were also able to more accurately evaluate the interaction ability of TCF4 fragments. For each interaction experiment, 60 transformant colonies were therefore assayed for growth on dropout medium plates lacking histidine and containing increasing doses of 3-aminotriazol (3-AT), an inhibitor of HIS3. With increasing doses of 3-AT, more and more of the enzyme is inhibited, selecting for stronger gene activation and more stable interactions (Figure 4C).

**Figure 5:**
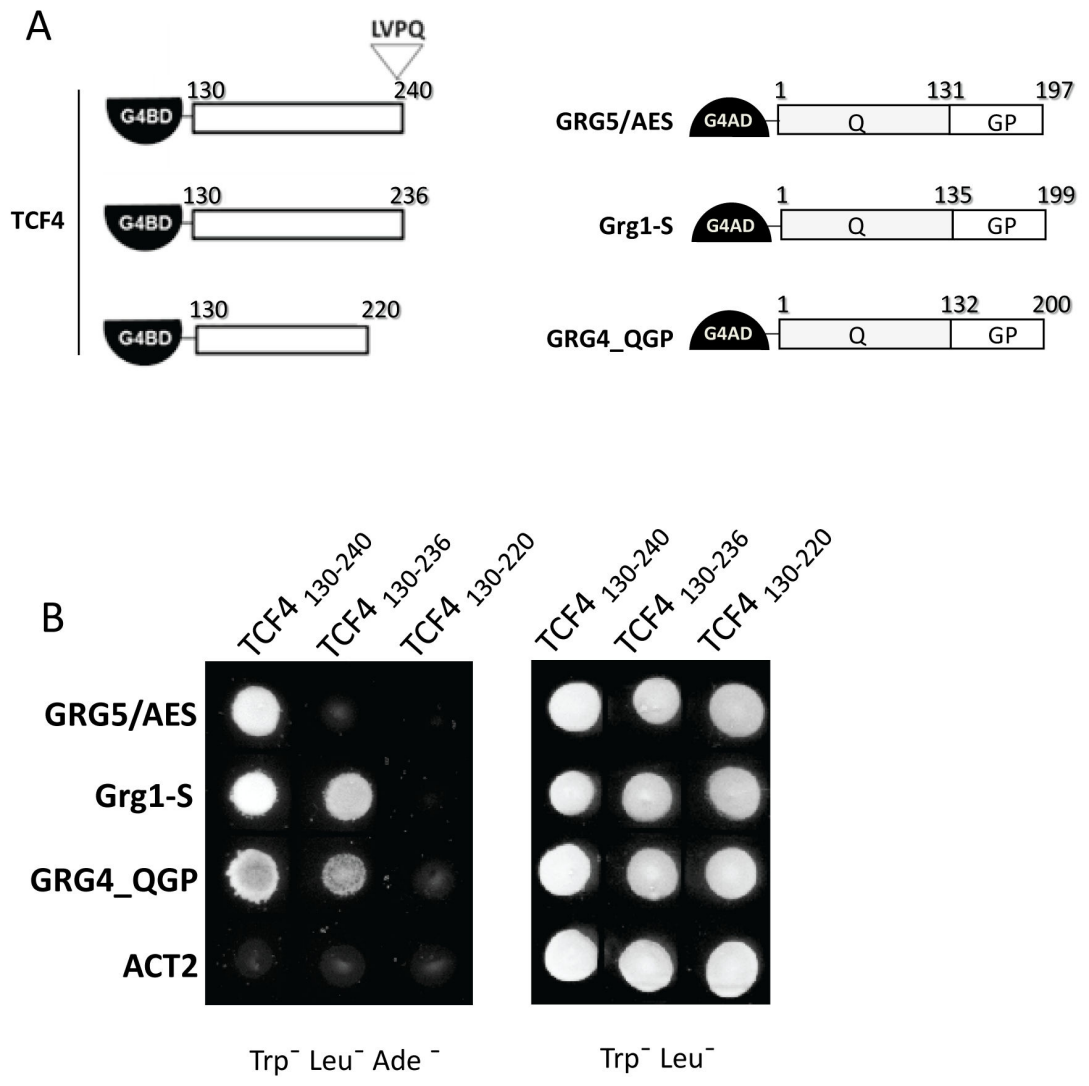
Convergent and divergent sequence requirements for TCF4 binding to different GRGs. A) Schematic view of TCF4 (left) and GRGs (right) fusion constructs used in yeast-two hybrid assays. B) Two-hybrid analysis of the importance of amino acids 130-240, 221-240 and the LVPQ motif for the interactions between TCF4 and GRGs. Co-transformant yeast cells were selected in medium lacking tryptophan and leucine and assayed for the indicated interactions using the adenine reporter gene (in Trp-Leu-Ade-medium). The same 111-amino acid region (residues 130-240) is sufficient for strong interaction with all three GRGs tested, and amino acids 221-240 are required for it, whereas removal of the LVPQ motif abrogates binding to GRG5/AES, but not the other GRGs, although it has some effect on the TCF4-GRG4 interaction. The ACT2 plasmid was used as a negative control.

Because the 253-FRQ/HPY-257 motif in TCFs has been suggested to be involved in binding to Groucho family members [48,83,84], we tested a TCF4 fragment lacking this pentapetide (TCF4_130-240_). As shown in Figure 4B and 4C, transformant yeast cells grew well, both in medium lacking adenine and in medium lacking histidine, even at the highest 3-AT concentration tested (30mM), demonstrating that the motif is not required for this specific interaction. In contrast, TCF4_130-220_ completely lacked GRG5/AES-binding (Figure 4B), showing that amino acids 220-240 are required for the interaction. We further determined that fragments 151-260 and 172-260 are still able to interact with GRG5/AES and activate the adenine reporter (Figure 4B). However, the binding affinity was reduced, as shown by the limited growth of transformants in medium lacking histidine at high 3-AT doses (Figure 4C). Additionally, TCF4_172-240_ showed dramatically reduced GRG5/AES-binding and the interaction was only observed under low stringency 3-AT selection (Figure 4C). Altogether, these data indicate that the GRG5/AES-interacting domain of TCF4 resides within amino acids 130-240 and that this region is sufficient for a strong TCF4-GRG5/AES interaction. Importantly, strong interaction of the TCF4_130-240_ fragment was also observed with Grg1-S and GRG4-QGP (compare Figures 4B and 5B), suggesting it defines a general core region for TCF4-GRG interactions.

### The LVPQ motif is critical for TCF4’s interaction with GRG5/AES but not with other Grouchos

The 20-amino acid segment of TCF4 we determined to be essential for GRG5/AES-binding (amino acids 221-240) encompasses the LVPQ motif, which is present only in some TCF4 splice variants, and invariantly found in TCF3 [81,82]. Because this motif has been implicated in repression mediated by these particular TCFs [81,82,85], we investigated whether it plays a role in TCF4-GRG interactions, by testing the effects of deleting it from TCF4_130-240_ in yeast two-hybrid assays (Figure 5). As shown in Figure 5B, absence of the LVPQ motif completely abrogates interaction with GRG5/AES, implying that it plays a critical role in TCF4-GRG5/AES binding. However, this cannot be generalized to other GRGs, as the LVPQ deletion had no effect on TCF4’s interaction with Grg1-S and only mildly hampered its binding to GRG4-QGP, a "truncated" GRG4 that, like GRG5/AES or Grg1-S, includes only the Q and GP domains (Figure 5B). These results show that Groucho proteins bind differently to TCF4 and suggest its interaction with GRG5/AES might be modulated by alternative splicing.

### GRG5/AES represses Wnt signaling in human cells and cooperates both with dnTCF4 and Grg1-L for efficient transcriptional repression

**Figure 6:**
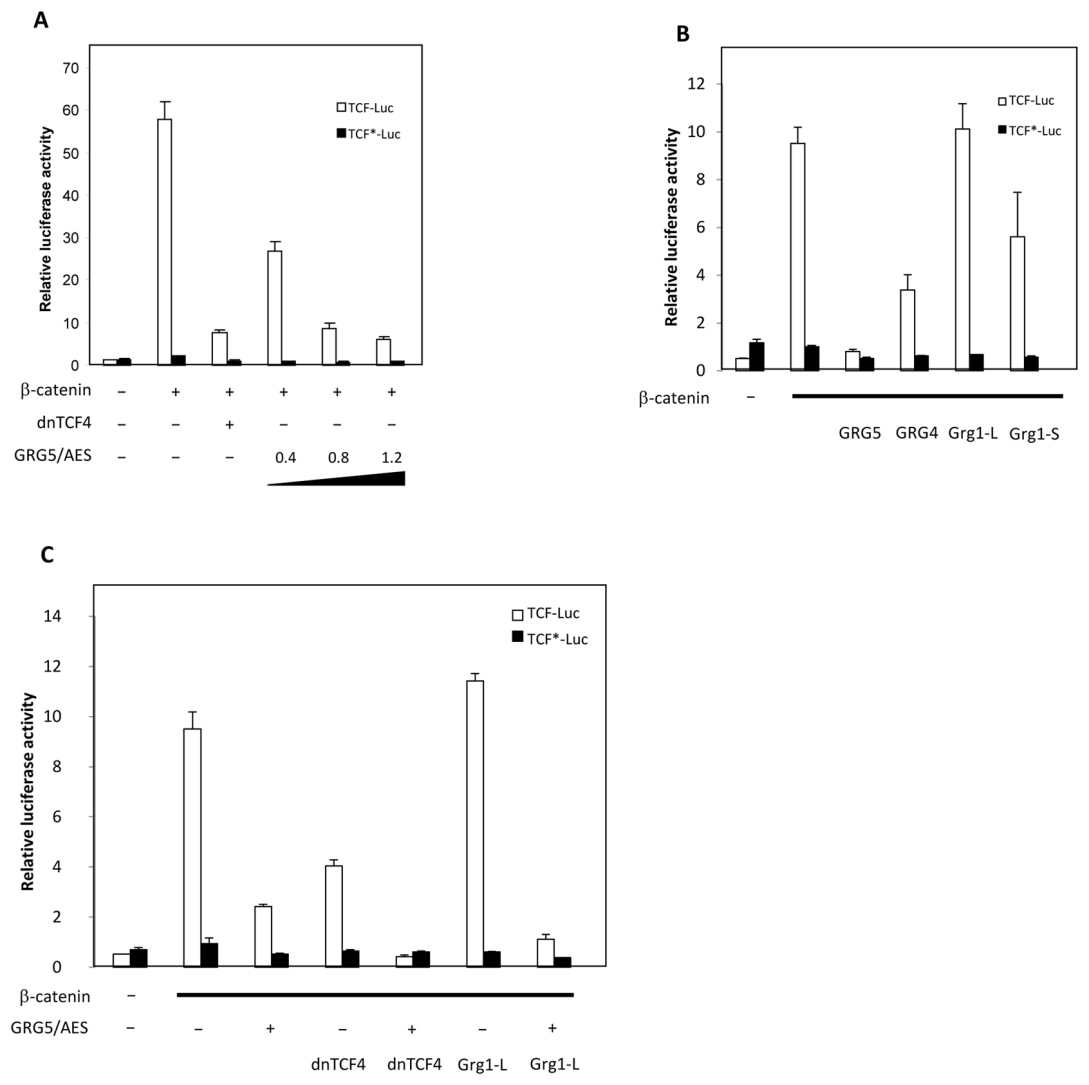
GRG5/AES represses TCF-β-catenin mediated transcription and co-operates with both dnTCF4 and Grg1-L for transcriptional repression. HEK293 cells were transfected with either the reporter construct TCF-Luc (containing three TCF consensus binding sites upstream of the firefly luciferase cDNA), or the control reporter TCF*-Luc (with mutations in the TCF-binding sites) and the indicated combinations of expression plasmids. A) Addition of GRG5/AES plasmid decreases the luciferase activity induced by transfected βcat and endogenous TCFs in a dose dependent manner. At the highest dose (1.2 μg/μl), GRG5/AES-mediated repression is comparable to the effect of dominant negative TCF4 (dnTCF4), a previous established repressor of the canonical Wnt pathway [13]. B) Repression of TCF-β-catenin mediated transcription by various Groucho-family proteins. In contrast to “long” Groucho Grg1 (Grg1-L), GRG5/AES, GRG4 and Grg1-S (a short GRG1 isoform [63]) effectively repressed TCF-dependent luciferase activity. 1.2 µg were used for transfection of each Groucho expression plasmid. C) Co-transfection of GRG5/AES with either dnTCF4 or Grg1L (0.4µg of each plasmid) results in higher repression of TCF-β-catenin dependent luciferase activity then transfections with each effector plasmid individually. β-galactosidase assays were performed as an internal control for transfection efficiency. All the assays were done in triplicate in three independent experiments.

GRG5/AES has variously been reported to act either as a dominant negative, that interferes with the function of the long GRGs [49,62] or, like these, as a co-repressor of multiple transcription factors, including members of the TCF family [66–68,86]. Together with our observation of an interaction between GRG5/AES and TCF4 this prompted us to examine the ability of GRG5/AES to counteract TCF-β-catenin-mediated transcription (Figure 6). As previously observed in other cell lines [71], co-transfection of HEK293 cell line with a TCF-Luc construct (containing three TCF consensus binding sites upstream of the firefly luciferase cDNA) together with β-catenin resulted in a remarkable increase of luciferase activity compared to TCF-Luc expression alone (Figure 6A). Addition of increasing amounts of *GRG5/AES* plasmid reduced luciferase reporter activation in a dose-dependent manner (Figure 6A). At the highest dose tested, GRG5/AES suppressed TCF promoter activity 12-fold, similar to the repressive effect of a dominant negative form of TCF4, a well-accepted antagonist of TCF-β-catenin signaling [18]. As expected, co-transfection with the control reporter TCF-Luc* (with mutated TCF binding sites) resulted in no alteration of luciferase activity.

To further evaluate the putative repressive function of GRG5/AES, we compared its activity to those of other Groucho-family members, broadly accepted as general co-repressors of the TCF/LEF-family of proteins: GRG4, Grg1-L and Grg1-S. As shown in Figure 6B, GRG5/AES was found to repress TCF-β-catenin-dependent transcription in human cells similarly to other family members tested - and indeed more efficiently than GRG4 (see also Figure S1). Surprisingly, in this specific cell line, the “long Grg1" (Grg1-L) was not capable of repressing transactivation of the TCF-Luc reporter gene (Figure 6B). This might have resulted from a lower protein expression level in the cells (Figure S1), or rather be another indication of the complex and context-dependent nature of Groucho proteins’ functions. To determine whether GRG5/AES is actively repressing TCF-dependent transcription as has been demonstrated for "long Grouchos" [87], or rather functioning by blocking the formation of TCF-β-catenin active complexes, we tested the ability of GRG5/AES to interfere with basal transcriptional activity when tethered to DNA. Gal4-fusion constructs encoding either GRG5/AES or full-length Grg1 (Grg1-L) were therefore transfected into HEK293 cells and their ability to repress the activity of a strong, Gal4-responsive, promoter was compared. As shown in Figure S2, GRG5/AES is able to repress basal transcription to the same degree as Grg1-L, implying that GRG5/AES harbors a functional repression domain comparable to those of the long Grouchos. These results demonstrate that GRG5/AES can effectively repress endogenous TCFs and down-regulate TCF-β-catenin-mediated transcription.

Because HEK293 cells express all TCF/LEF-family members [62], we could not determine whether this repression was mediated through TCF4. To address this limitation, we co-transfected HEK293 cells with both GRG5/AES and dnTCF4 expression plasmids, to test whether they act cooperatively or independently in repressing TCF-Luc reporter transactivation. As shown in Figure 6C, this resulted in 22.5-fold transcriptional repression, significantly higher than the 6.3-fold additive effect of GRG5/AES- and dnTCF4-mediated repressions. This implies that GRG5/AES and dnTCF4 indeed act cooperatively in transcription repression, suggesting that the TCF4-GRG5/AES interaction contributes to GRG5/AES’s repressor function. Furthermore, we asked whether GRG5/AES could similarly cooperate with Grg1-L, using the same reporter gene assay (Figure 6C). Interestingly, although Grg1-L alone was unable to repress TCF-mediated transactivation in this assay, its co-transfection with GRG5/AES significantly increased the latter’s repression activity, showing that GRG5/AES can cooperate with the "long Grouchos" in downregulating Wnt/β-catenin signaling.

### GRG5/AES counteracts β-catenin dependent dorsal specification in zebrafish

In zebrafish, as in *Xenopus* , nuclear accumulation of β-catenin in the early embryo is required for activation of dorsalizing genes and establishment of the dorsal-ventral (DV) axis [56,88,89]. Furthermore, both β-catenin and Wnt overexpression result in embryo dorsalization and secondary axis formation [14,20,54,90–92]. Our observations that GRG5/AES interacts with TCF4 and behaves as a consistent repressor of TCF-β-catenin dependent transcription in human cells therefore raised the possibility that GRG5/AES may also regulate DV patterning during early zebrafish embryogenesis.

**Figure 7:**
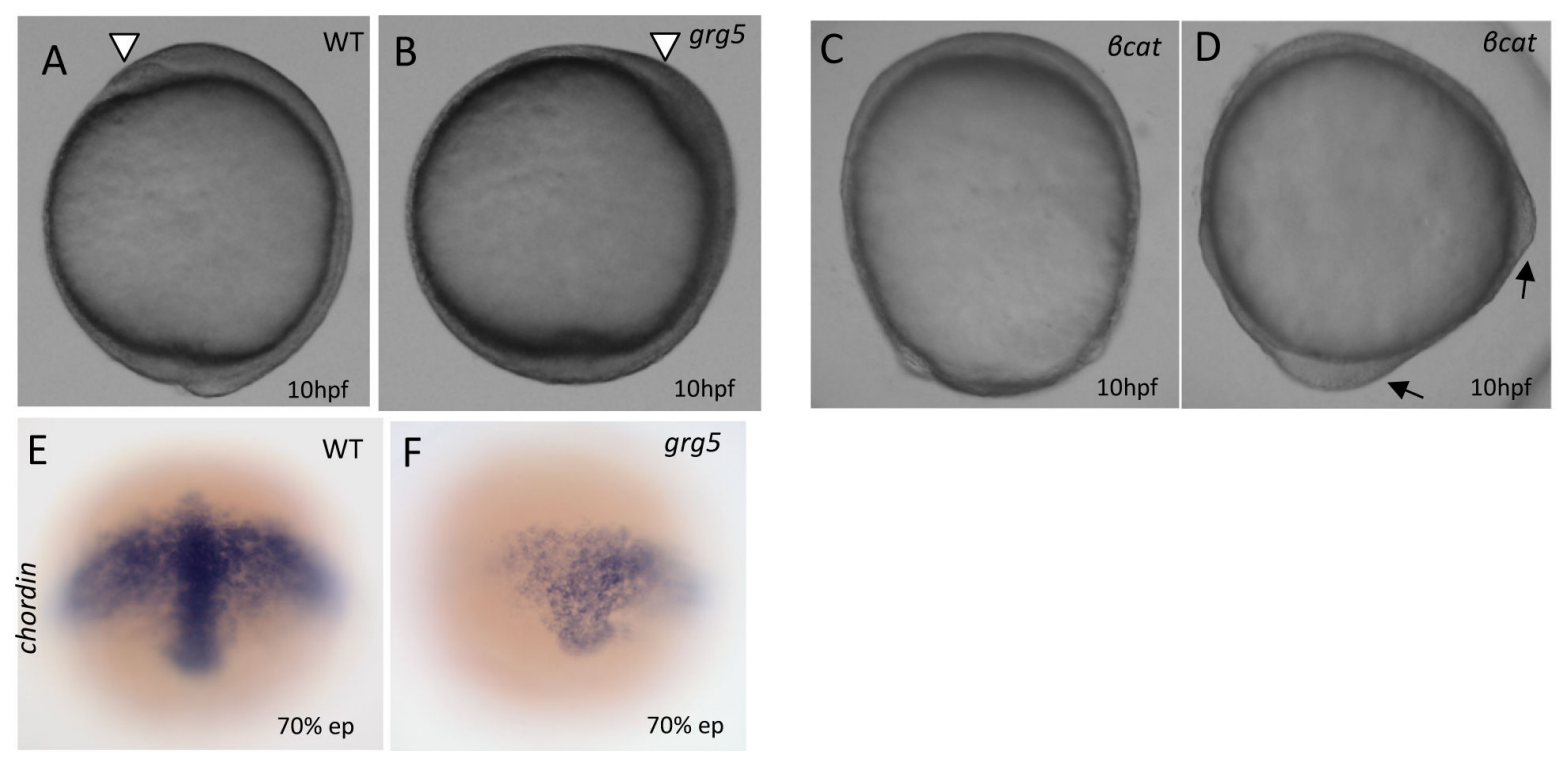
GRG5/AES overexpression leads to ventralization of zebrafish embryos. (A–D) Lateral view, dorsal to the right, of 10hpf-stage embryos. (A) Control uninjected; (B) Injected with 250pg of *grg5/aes* mRNA; arrowhead: anterior end of the neural plate with formation of the “polster” in the prospective head region; (C–D) Injected with 100pg of an activated *β-catenin* mRNA. Black arrows: ectopic dorsal axis; E-F) Whole-mount in situ hybridization for *chordin* expression at 70% epiboly uninjected (E) and *grg5/aes*-injected (F) embryos.

To test for this possibility, we started by microinjecting *grg5/aes* mRNA into 1-cell stage embryos (Figure 7). Morphologically, the vast majority of injected embryos were apparently normal at 10hpf, with the characteristic thickening of the neural plate along the dorsal side. However, some embryos displayed reduction of the dorsal side (Figure 7B). In contrast, and as previously reported, overexpression of an active β-catenin (T41A) resulted in dorsalized embryos, easily recognized by an animal-vegetal elongated morphology and, in some embryos, formation of ectopic dorsal axes [54] (Figure 7C, D). To further characterize the effect of *grg5/aes* microinjection, we investigated the expression pattern of the dorsal marker *chordin* by performing *in situ* hybridizations at mid-gastrulation in *grg5/aes*-injected embryos. As shown in Figure 7, *chordin* expression was clearly reduced, suggesting that GRG5/AES indeed influences DV axis patterning by inhibition of dorsal-specific gene expression.

**Figure 8:**
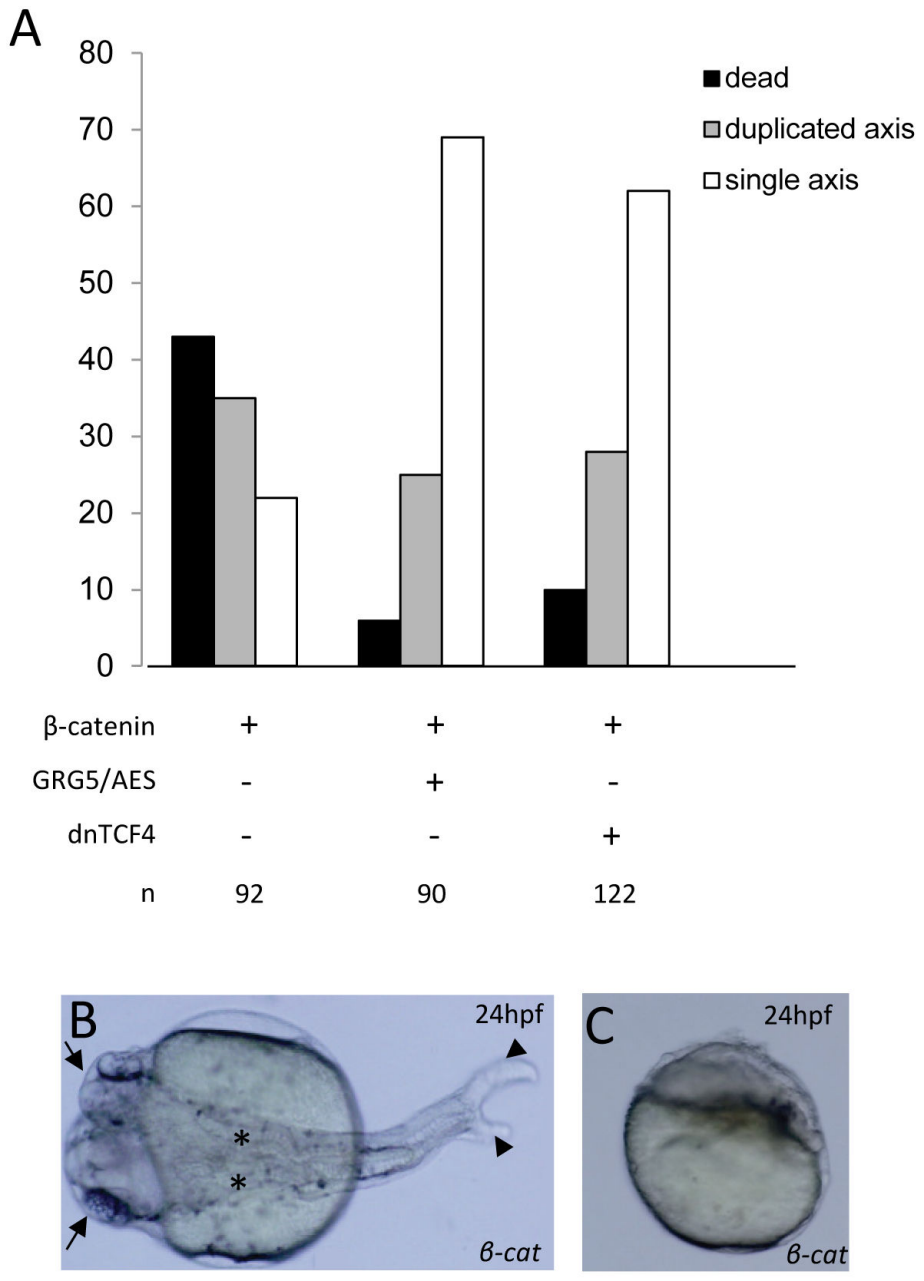
GRG5/AES reduces both mortality and axis duplication in β-catenin-overexpressing embryos. A) One blastomere of 8-cell stage zebrafish embryos was injected with the indicated combinations of *β-catenin* (50 pg), *grg5/aes* (500 pg) and *dntcf4* (500 pg) mRNAs. Partial and complete axis duplications were grouped to avoid ambiguity. The total number of scored embryos (n) is indicated at the lower part of the panel. B) Example of 24hpf embryo showing complete axis duplication (induced by β-catenin injection), with two discernible heads (arrows), notochords (asterisks) and tails (arrowheads). Dorsal view, anterior to the left. C) Example of 24hpf embryo injected with *β-catenin* showing extreme dorsalization.

Next, we sought to evaluate GRG5/AES’s ability to antagonize β-catenin dorsalizing activity in zebrafish embryos. When we microinjected *β-catenin* into a single blastomere of 8-cell stage embryos, severe disruption of DV patterning occurred. Forty-five percent of injected embryos were dead at 24hpf, likely as a consequence of extreme dorsalization (Figure 8), while 62% of the remaining embryos displayed axis duplication. While a complete secondary axis was obtained on a few occasions, partial axis duplications were more common. Nevertheless, to avoid ambiguity, we grouped both types in the same class, scoring embryos as having double-axis whenever there was notochord duplication (Figure 8B). In contrast, co-injection of *β-catenin* and *grg5/aes* mRNAs into a single blastomere of 8-cell stage embryos resulted in a much higher survival rate (Figure 8A). The number of embryos dead at 24 hours was reduced 9 fold, and dorsalized phenotypes were more moderate compared with those produced by *β-catenin* injection alone (data not shown). Furthermore, there was a consistent decrease in the number of embryos with a duplicated axis, indicating that GRG5/AES antagonizes β-catenin signaling in zebrafish embryos. Comparable results were observed in embryos co-expressing β-catenin and dnTCF4 (Figure 8A), a truncated version of TCF4 that lacks the β-catenin binding domain and is similar to constructs previously shown to inhibit axis formation in both *Xenopus* and zebrafish [13,14].

**Figure 9:**
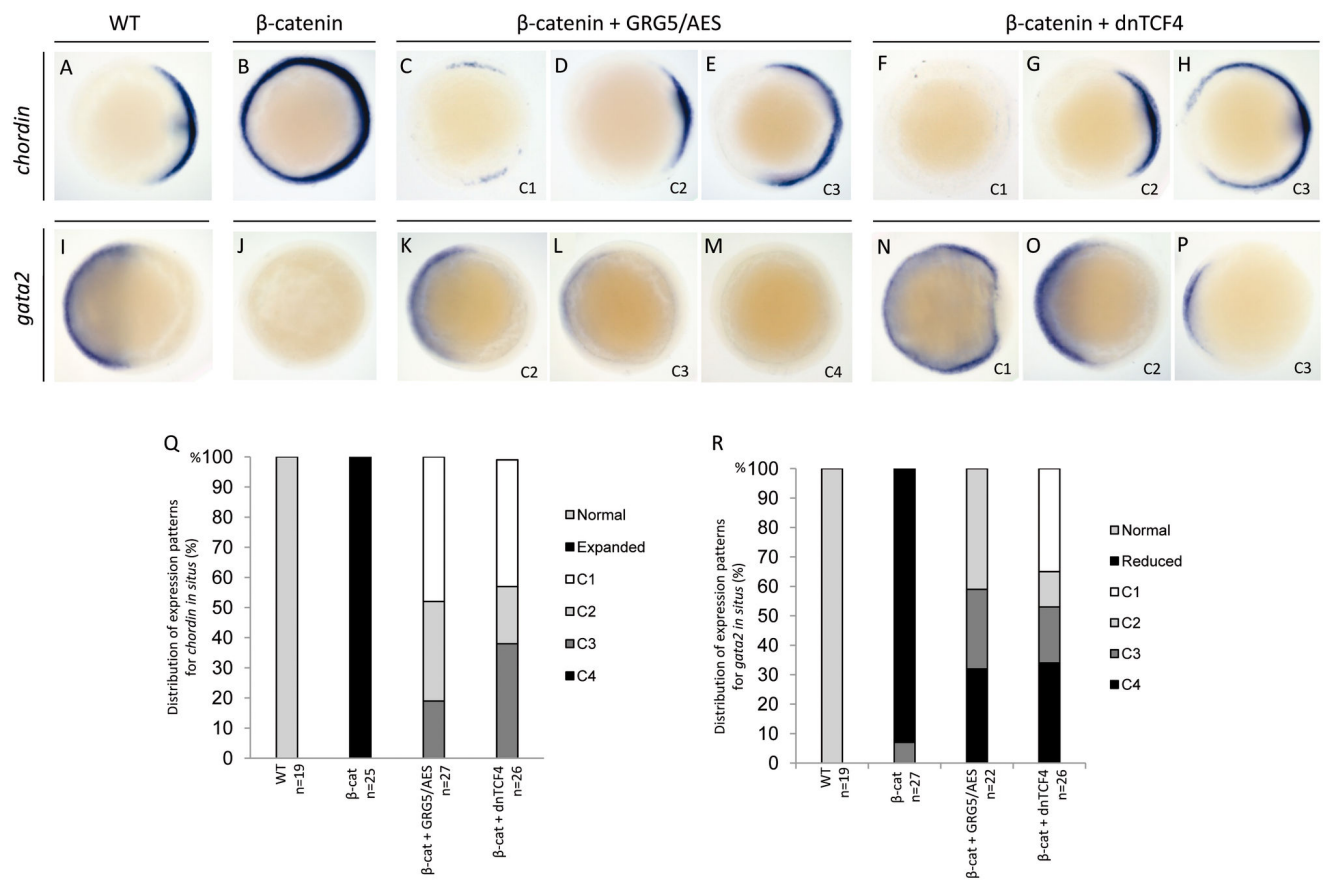
GRG5/AES antagonizes β-catenin in dorsal-ventral patterning during early zebrafish embryogenesis. Whole-mount *in situ* hybridizations for *chordin* (A–H) and *gata2* (I–P) of double-injection experiments (columns). Animal pole views, dorsal to the right, of 70% epiboly stage-embryos. Injection of *β-catenin* strongly dorsalizes embryos, as shown by an expanded and circumferential expression of the dorsal-specific gene *chordin* (B) and a total absence of the ventral-specific gene *gata2* (J). Co-injection of *grg5/aes* (C-E; K-M) rescues β-catenin-induced dorsalization and restores normal expression domains for both marker genes. *dntcf4* co-injections (F–H; N–P) were used as a control for β-catenin antagonism. Co-injected embryos were grouped in four classes of marker gene expression: C1 (C, F) – abnormal expression consistent with embryo ventralization; C2 (D, G; K,O) − restoration of normal expression patterns (A,I); C3 (E, H; L, P) – partial reversion of β-catenin-induced abnormalities; C4 (M) − abnormal expression consistent with embryo dorsalization. Q–R: distribution of the embryos in the different classes of *chordin* (Q) and *gata2* (R) expression. GRG5/AES antagonizes β-catenin effects on *chordin* and *gata2* in 100% and 68% of the embryos respectively. n: number of embryos tested.

The ability of GRG5/AES to counteract β-catenin *in vivo* was further explored by examining the expression of markers for the DV character of the injected embryos (Figure 9). During gastrulation, *chordin* is expressed in the prospective dorsal region of the embryo [75] and, by 70% epiboly, it encompasses approximately half of the circumference of the margin (Figure 9A). Conversely, *gata2* expression is restricted to the ventral region (Figure 9I). As expected, dorsalized *β-catenin*-injected embryos displayed completely circular *chordin* expression in all of the tested embryos (Figure 9B,Q) and, consistently, a complete suppression of *gata2* (Figure 9J, R). However, when *grg5/aes* mRNA was co-injected, β-catenin-induced dorsalization was efficiently rescued as shown by restoration of normal marker gene expression patterns. The embryos were placed into four classes based on DV extent of marker gene expression (Figure 9Q). All the co-injected embryos tested for *chordin* showed reduction of the circular expression induced by β-catenin. In 48% of the embryos, the *chordin* expression domain was restricted to less than 40% of the margin cells or was completely absent (class 1, Figure 9C, Q), indicating loss of dorsal-specific gene expression caused by GRG5/AES. In 33% of the embryos tested, the normal pattern of *chordin* expression was restored (class 2, Figure 9D, Q); 19% still showed ventrally expanded expression when compared to uninjected controls, but to a lesser degree than embryos injected only with *β-catenin* (class 3, Figure 9E, Q). GRG5/AES was also able to rescue *gata2* expression in 68% of the total embryos, either fully (41%) or partially (27%) (Figure 9I–M, R). Similar results were observed in control experiments of β-catenin and *dntcf4* co-injections, with clear suppression of β-catenin induced dorsalization and strong evidence of a ventralizing effect (Figure 9F–H, N–P).

### GRG5/AES antagonizes Wnt/β-catenin signaling during early zebrafish embryogenesis

**Figure 10:**
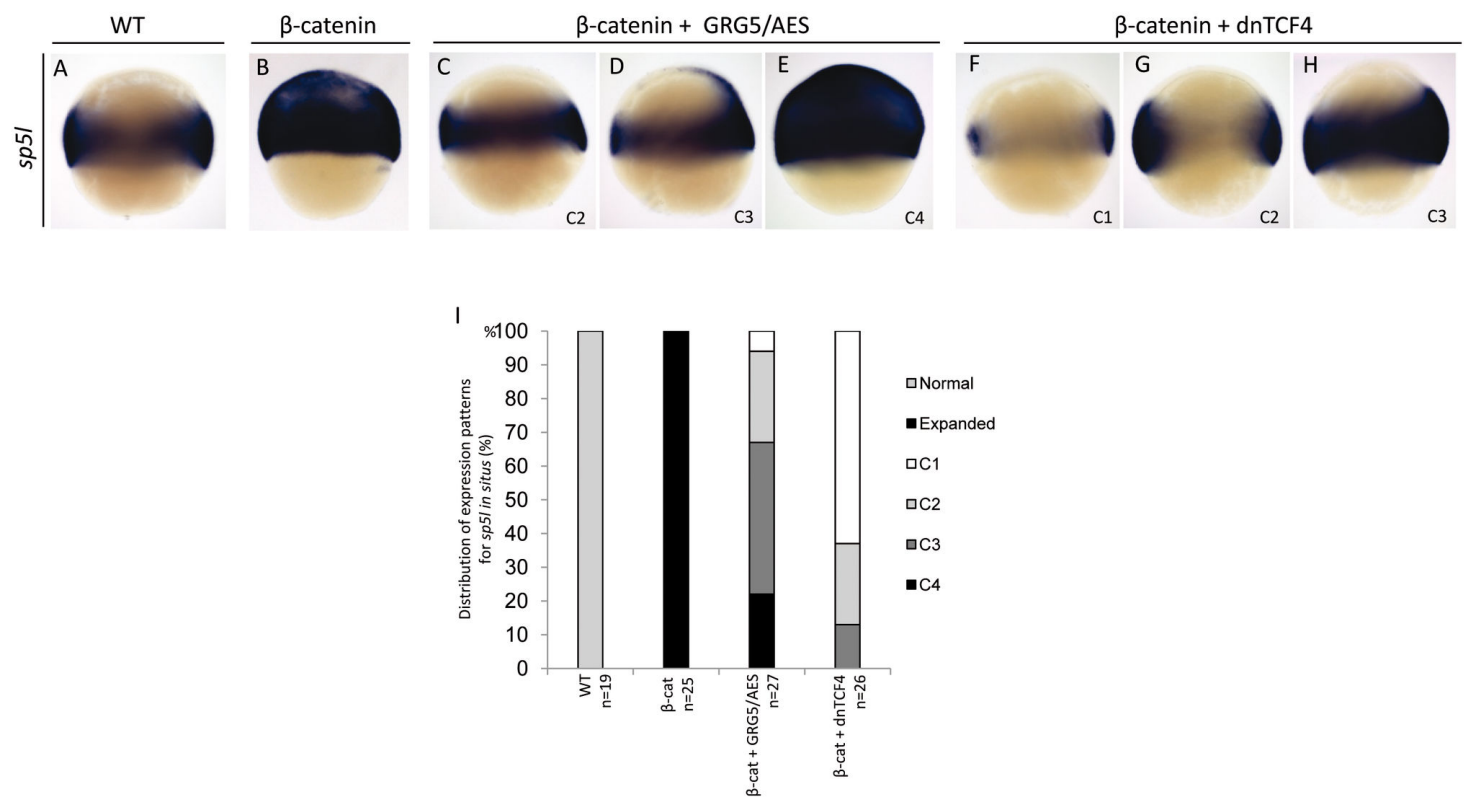
GRG5/AES represses Wnt signaling during early zebrafish embryogenesis. Whole-mount *in situ* hybridizations for *sp5l*, a direct target of canonical Wnt signaling. Lateral view, dorsal to the right, of 70% epiboly stage-embryos. Injection of *β-catenin* induces broad ectopic domains of *sp5l* expression (B), which are reversed by co-injection of *grg5/aes* (C–E) or *dntcf4* (F–H). Expression patterns in co-injected embryos were grouped in four classes, as above. AES reversed β-catenin-induced misexpression in 75% of the embryos (I). n: number of embryos tested.

*Sp5l* is a direct target of canonical Wnt signaling whose expression reflects Wnt involvement in mesoderm and neuroectoderm patterning during gastrulation [93,94]. We therefore predicted that, if indeed GRG5/AES antagonizes β-catenin activity, it should be able to rescue, at least partially, *sp5l* upregulation induced by β-catenin. As expected, injection of activated *β-catenin* mRNA resulted in greatly expanded *sp5l* expression at the animal pole of 70% epiboly embryos (Figure 10B) and this overexpression was clearly reduced upon co-injection of *grg5/aes* mRNA. The embryos analyzed by in situ hybridization were classified as above, in different classes of rescue efficiency and a representative example of each class is shown (Figure 10C–E). In 75% of the co-injected embryos, GRG5/AES repressed β-catenin-induced overexpression, from which 45% were partially rescued, while in 27% the normal expression pattern was completely restored. Co-injection of *dntcf4* control mRNA similarly suppressed or completely abolished *sp5l* overexpression (Figure 10I).

## Discussion

### TCF4 binds to GRG5/AES in yeast and human cells

Despite intensive work to describe how the TCF-β-catenin pathway operates in a variety of developmental processes, a detailed understanding of the specific molecular mechanisms that ﬁne-tune basic signaling is still lacking. Although β-catenin regulation has been extensively studied, much less is known about the regulation of TCFs and how individual members of this family dictate Wnt signaling output in different cell types. We were particularly interested in TCF4 because it plays pivotal roles in a variety of processes, including central-nervous system patterning [95,96], skin homeostasis and wound repair [97] and particularly normal development and pathology of the intestine [17,18,29,98]. We therefore performed a yeast two-hybrid screen for new putative TCF4-interacting proteins and demonstrated that TCF4 physically interacts with GRG5/AES, a distinct member of the GRG family that exemplifies a subclass of “truncated” proteins, commonly referred to as the “short Grouchos”. This observation was unexpected, because it had been previously reported that mouse Grg5 did not interact directly with TCF4, although it did with other TCFs [49]. Interestingly, in the same study, mouse Grg5 was shown to interact with *Xenopus* TCF3 but not with the orthologous mouse protein. This might be due to sequence divergence among different species, affecting binding specificities. However, there is a growing body of evidence for the existence of multiple isoforms of both GRG5/AES and TCF4, resulting from extensive alternative splicing and/or alternative promoter usage [79,82,99,100]. As would be expected from first principles, and has been confirmed by several reports, this structural variation results in specificity in protein–protein interactions, with profound functional implications [81,99,101]. It is therefore possible that apparently conflicting results stem from the use of different isoforms, as it is not always clear in the literature which particular one was used. At any rate, our work clearly demonstrates the existence of an interaction between TCF4 and GRG5/AES, confirmed by co-immunoprecipitation and transcription repression assays in human cells. This result is particularly important because it was obtained in an unbiased way – in a cDNA library screen for TCF4 interacting proteins where GRG5/AES was the most frequently obtained candidate – and not through candidate testing. Additional support for an *in vivo* interaction between TCF4 and GRG5/AES comes from the overlapping expression patterns of these two proteins observed in several vertebrates [86,102–105] and the evidence for their critical roles during pituitary gland development [102].

### Isoform-specific regions of TCF4 are critical for differential binding to GRGs

All TCF/LEF family members share two highly conserved structural features: a 55-amino acid N-terminal domain responsible for β-catenin binding and a centrally located high-mobility-group (HMG) box that mediates DNA-sequence recognition and bending for an architecturally favourable context for transcription control [2–4] (Figure 1A). Between these two domains is one of the most divergent regions among the family members (and even orthologues), to which various roles involving protein–protein interactions have been assigned [43,46,106,107]. GRG binding has also been broadly assigned to this region in previous interaction studies for TCF1, *Xenopus* TCF3 and LEF1, though recently a second (and weaker) site in LEF1’s HMG box has been described as mediating interaction with *Drosophila* Groucho [69]. No interaction mappings were previously reported for TCF4 and, due to the low sequence conservation in this region, results previously obtained with other TCFs are not easily transposable to TCF4. We performed an exhaustive mapping of the GRG5/AES-binding region in TCF4 by yeast two-hybrid and determined that a 111-amino acid stretch (residues 130-240), located between the β-catenin binding domain and the HMG box, is sufficient for the TCF4-GRG5/AES interaction (Figure 4). Interestingly, this region largely corresponds to an alternative-splicing prone region, encompassing exons 4-6, only present in some TCF4 variants [99,101].

Although this fragment displays some similarity to the GRG-binding regions described for other TCFs (Figure S3), no highly conserved sequences are recognizable, in contrast to what has been reported for other GRG partners, which bind through specific and short motifs [108]. Indeed, it had been speculated that the pentapeptide FRQ/HPY present in TCF/LEF-family members might be involved in the interaction with GRGs [48,83] because it is somewhat similar to the motifs found in other GRG partners, such as Hairy-related proteins (WRPW) [109], Runt (VWRPY) [110] and HKb (FRPW) [84], but we have demonstrated that this motif is not required for binding between TCF4 and GRG5/AES (Figure 4). Equally dispensable is the sequence corresponding to a short LEF1 stretch proposed to mediate Groucho binding (Figure S3) [69].

Our mapping refinement assays further led us to identify both convergent and divergent requirements for TCF4 binding by various GRGs. Indeed, the 111-amino acid core region for the TCF4-GRG5/AES interaction was also shown to be sufficient for TCF4 binding to GRG4 and GRG1. Similarly, the last 20 residues (221 to 240) of this region were found to be critical for all three interactions, as their removal abrogated them. On the other hand, GRG5/AES, but not GRG1 or GRG4 binding depends on the LVPQ motif, which is located in the 221-240 region and, within the TCF family, is only present in TCF4 as a result of specific alternative splicing, and invariantly in TCF3 (Figure S3). The LVPQ motif had previously been implicated in repression mediated by these particular TCFs [81,82], but its role in modulating TCF-GRG interactions was unknown. Our observations therefore support the idea that binding of GRG5/AES depends on the expressed splice TCF4 variant and that contradictory results may be explained by different splice variants of TCF4 used in different reports. More importantly, it strengthens the idea that multiple TCF4 isoforms can differentially influence tissue-specific Wnt responses.

Nevertheless, it should be noted that an additional segment (amino acids 130-171) is also required for binding. This might indicate that TCF-GRG interactions, rather than being mediated by a single short motif, involve multiple cooperative binding events. Alternatively, it is possible that more important than specific sequences is a favorable conformation of the GRG-binding region to which the various segments found to be required contribute. Consistent with this possibility, though GRG family members bind the same TCF/LEF proteins, different specific requirements seem to underlie individual interactions, the only obvious feature shared by the GRG-binding regions in TCFs being a high proline content (Figure S4). Interestingly, similar proline-rich regions have been identified in other transcription factors that interact with Groucho-related proteins and are also a feature of several transcription repressors [65,67,111–113]. The significance of this observation is unknown, but it might point to a requirement for a disordered structure of the TLE/GRG-binding regions.

### GRG5/AES represses TCF-β-catenin-mediated transcription in human cells and synergizes with Grg1-L for efficient repression

The long Grouchos are generally recognized as dedicated co-repressors of a wide range of DNA-binding factors, including the TCF/LEF family [59,108,114,115]. The highly conserved Q-domain mediates interaction with TCF/LEF transcription factors, as well as tetramerization, which is thought to be a prerequisite for repressor function [116–118]. Furthermore, the GP domain interacts with histone deacetylases (HDACs) in repressor complexes, promoting a more compact and transcriptionally inactive chromatin [47,62,119,120]. As for the short Grouchos, and particularly GRG5/AES, the situation is more complex. Similarly to the long proteins, human GRG5/AES was originally described as a transcriptional co-repressor [67] but subsequent reports have provided evidence that it can act either as a co-repressor [67–69,86,121,122] or as a “de-repressor” [62,116,117]. Many of the interactions between the long GRGs and different transcription factors are mediated by their C-terminal WD domains (Figure 1) and in these cases a mechanism for de-repression can easily be postulated: a short Groucho, incapable of interacting with the co-repressor protein but still able to bind other Groucho family members, might “poison” tetramers, by rending them less proficient at forming repression complexes [65,112,113]. In the case of TCFs, however, the interaction is mediated by the Q domain [62], present in both the short and long Grouchos, making this mechanism unlikely. Initial reports of GRG5/AES as a TCF de-repressor therefore required an alternative explanation: the divergence between the GP domains of the long GRGs and those of GRG5/AES make the latter unable to interact with HDACs 1 and 3. However, this explanation is still not satisfactory for two reasons. First, lack of HDAC binding does not prevent GRG5/AES from acting as a co-repressor for other transcription factors [66–68,86], possibly through interaction with the basal transcription machinery. Second, structural studies have suggested that GRG-mediated repression of TCF-β-catenin dependent transcription is based on competition for TCF binding – at which GRG5/AES is fully proficient [123].

In this study, we demonstrate that GRG5/AES consistently behaves as a TCF co-repressor, in both human cells and zebrafish embryos. More specifically, we show that, at least in HEK293 cells, GRG5/AES is able to strongly repress TCF-β-catenin transcription in a dose-dependent manner. Additionally, we observed a similar, though less pronounced, repressive effect of GRG5/AES in DLD-1, a colorectal tumor cell line which displays constitutive TCF4-β-catenin dependent transcription due to APC mutations (data not shown). This is in agreement with a study published during the preparation of this manuscript, showing that GRG5/AES is also able to repress LEF1 in human cell lines [69], but in stark contrast to what was observed in another recent study, in which it de-repressed LEF1-mediated activity [124]. Moreover, our work shows that GRG5/AES is also able to synergize with long Grouchos for strong TCF-mediated repression, thus providing additional support to the growing idea that GRG5/AES is not a general GRG antagonist and highlighting the importance of taking caution with generalizations about a protein’s function and biological effects.

How can these apparently conflicting observations regarding GRG5/AES’s role in repressing TCF-β-catenin dependent transcription be reconciled? Our studies strongly suggest that GRG5/AES’s function is critically dependent on the specific TCF/LEF isoforms present in the cell. For example, as previously noted, the TCF4 region involved in GRG5/AES binding is affected by alternative splicing, which could, in some cells, give rise to isoforms that do not interact with GRG5/AES. A similar mechanism is behind TCF1’s role as a Wnt antagonist in the intestine: because the prevailing TCF1 isoform in that tissue lacks a β-catenin interacting region, it can only form Wnt-signaling repressing complexes and therefore acts as an intrinsic dominant-negative [125]. The relative abundances of other GRG interactors might also be important. Indeed, some of them are well known to interact only with the long Grouchos, and it is also possible that others have a preference for the short ones, like GRG5/AES. The balance of all these partners in a cell is therefore likely to affect GRG5/AES’s availability to functionally interact with TCFs. Interestingly, the same process could also limit the availability of one or another of the long Grouchos, implying that, under specific circumstances, some of these might also behave as dominant negatives and potentially explaining our observation that Grg1-L, by itself, was unable to act as a TCF co-repressor in HEK293 cells (Figure 6B,C).

In both cases – differences in TCF isoforms and differences in other partners – a de-repressor role for GRG5/AES hinges upon the notion of “tetramer-poisoning” by a functionally impaired protein. This is consistent with the notion that homotetramerization is essential for efficient repression [121,126], but opposed by our findings of cooperation between GRG5/AES and Grg1-L and at least by another study showing that a *C. elegans* GRG5/AES-related protein was found to cooperate with a long Groucho orthologue in co-repression [122]. Alternatively, GRG5/AES’s subcellular distribution might be a crucial determinant of its apparently inconsistent behavior. As it varies in different cell contexts [124], it is conceivable that a predominantly cytoplasmic distribution could contribute to retain other GRGs in the cytoplasm, thus limiting their repressive activity.

### GRG5/AES represses dorsal cell fate in zebrafish and antagonizes Wnt signaling during DV patterning

Analysis of both mutant lines and overexpressing transgenics have demonstrated the indispensable role of canonical Wnt signaling in establishing the DV axis during early zebrafish embryogenesis [14,54,57,88,127–130]. Asymmetric nuclear accumulation of maternal β-catenin demarcates the specification of dorsal identity in the early embryo [56]. Parallel to what is observed in *Xenopus* , overexpression of β-catenin leads to strongly dorsalized embryos and formation of double axes [54,89]. In a similar way, and with the intrinsic limitations of all overexpression experiments, our results suggest that GRG5/AES might modulate DV patterning in the early embryo: first, GRG5/AES overexpression inhibits dorsal cell fate, effectively reducing endogenous expression of early dorsal-specific genes like *chordin*. Second, it can antagonize and rescue the most severe β-catenin-induced dorsalization phenotypes, including double-axis formation and expansion of dorsal domains (accompanied by a reciprocal reduction of ventral regions). Third, similar to a dominant negative version of TCF4, it downregulates expression of direct Wnt target genes and interferes with axis specification, most likely through TCF-GRG repressor complexes. These results consistently suggest that GRG5/AES can act as a repressor of Wnt signaling during early zebrafish development and concur with previous evidence for short Grouchos’ repressive activity *in vivo*. For example, in sea urchin, fragments of LvGroucho containing only the Q and GP domains (like GRG5/AES) or even the Q domain alone, were shown to inhibit TCF-β-catenin-mediated endomesoderm specification along the animal-vegetal axis [64]. Also, a naturally truncated version of GRG1/TLE1 was shown to counteract β-catenin in *Xenopus* [63] and, more recently, a GRG5/AES-related protein was found to act as a co-repressor to control developmental decisions in *C. elegans* [122].

Considering its endogenous expression in several species [86,108,131–134], including zebrafish (*Costa A. M. S. et al, manuscript in preparation*), we propose that GRG5/AES orthologues act endogenously as negative regulators of Wnt signaling during early patterning of the DV axis in the zebrafish embryo. It would be interesting, for example, to analyze if there is an upward gradient of TCF-GRG5/AES complexes along the DV axis, that mirrors the reported downward TCF-β-catenin gradient [88]. Also, given that some Tcf4 variants are maternally and zygotically expressed during zebrafish embryogenesis [104], similarly to what we found for zebrafish *Grg5* (*Costa A. M. S. et al, manuscript in preparation*), it is conceivable that specific Tcf4 isoforms may play a role in DV patterning. In fact, in *Xenopus* it was demonstrated that depletion of maternal XTcf4 resulted in reduction of *chordin* expression and ventralized embryos consistent with loss of organizer gene expression [135]. However, such roles for TCF4 have been difficult to definitively establish, as no phenotype was obvious in the available TCF4 mutants or knockdowns studies in several species [17,19,136], perhaps due to partial functional redundancy with other TCF-family members. In this context, a *Tcf4* zebrafish mutant lacking the LVPQ domain, which we found to be specifically needed for GRG5/AES-binding, could provide an invaluable tool to future studies of the physiological role of TCF4-GRG5 interaction during DV patterning and to gain further insight into how individual TCF4 splice variants may be involved in this process. An additional question is whether GRG5/AES plays an important role in the intestinal physiology of vertebrates. Since TCF4 is the most prominently expressed TCF/LEF family member in the gut, our characterization of the TCF4-GRG5/AES interaction may have important implications for normal intestinal development and tumorigenesis.

## Supporting Information

Figure S1 **All Grgs tested were highly expressed in HEK293 cells.**
HEK293 cells were transiently transfected with the Grg expression plasmids indicated above the lines, and 25 μg of total protein was loaded for Western-blot analysis, using either anti-HA or anti-Myc antibodies. All transfections resulted in high levels of expression for the corresponding proteins, but quantitative comparisons are limited by the use of different epitopes. The asterisk indicates a nonspecific band around 80 KDa, detected by the anti-HA antibody. Membranes were reprobed with an antibody specific for α - tubulin, which served as a loading control (lower blot).
(PDF)

Figure S2 **GRG5/AES has intrinsic repressive activity when directly bound to DNA.**
HEK293 cells were transfected with the 15UTSV-Luc reporter alone or together with the indicated effector plasmids. 15UTSV-Luc has high basal activity (resulting from the presence of an SV40 promoter) and is Gal4 responsive (because it contains 15 UAS_G_). Reporter activity repression by a Gal4-binding domain-GRG5/AES fusion (G4BD.GRG5) is similar to that of its Gal4-Grg1-L counterpart. An expression vector encoding only the Gal4-binding domain (G4BD) was used as a negative control.
(PDF)

Figure S3 **Amino acid alignment of human TCF/LEF family members.**
Amino acid numbers for each protein are indicated on the right. Black shading indicates identity, grey shading indicates similar residues. The two highest regions of homology are the N-terminal β-catenin binding domain (BD) (amino acids 1-53 of TCF4) and the HMG-box (amino acids 318-409 of TCF4). The GRG5/AES-interacting region in TCF4 mapped in this study (red box) corresponds to a 111 amino-acid stretch in the most variable region among TCFs (amino acids 130-240 of TCF4). The previously described [49] GRG/TLE-binding domain in TCF1 (amino acids 176-359 of TCF1) is delimited by asterisks (*). Blue boxes highlight three specific domains: LVPQ (amino acids 237-240 of TCF4) contained in the GRG5/AES-interacting region of TCF4; FRHPY (amino acids 253-257 of TCF4), not necessary for this interaction, and FPPHMV (amino acids 270-275 amino acids in TCF4), corresponding to the aligned region of a previously characterized domain involved in Groucho-binding for LEF1 [69], but not necessary for TCF4-GRG5/AES interaction. Sequences were aligned using the Clustal W algorithm.
(PDF)

Figure S4 **GRG-binding regions in TCF/LEFs display high proline content.**
Alignment of the 111-amino acid (amino acids 130-240) human TCF4 stretch to which we mapped GRG5/AES-binding to the other TCF/LEF human protein sequences using the Clustal W algorithm running on the Geneious v 5.3 program.
(PDF)

## References

[1] Castrop J, van Norren K, Clevers H (1992) A gene family of HMG-box transcription factors with homology to TCF-1. Nucleic Acids Res 20: 611. doi:10.1093/nar/20.3.611. PubMed: 1741298. 1741298 10.1093/nar/20.3.611 PMC310434

[2] Giese K, Amsterdam A, Grosschedl R (1991) DNA-binding properties of the HMG domain of the lymphoid-specific transcriptional regulator LEF-1. Genes Dev 5: 2567-2578. doi:10.1101/gad.5.12b.2567. PubMed: 1752444. 1752444 10.1101/gad.5.12b.2567

[3] Giese K, Cox J, Grosschedl R (1992) The HMG domain of lymphoid enhancer factor 1 bends DNA and facilitates assembly of functional nucleoprotein structures. Cell 69: 185-195. doi:10.1016/0092-8674(92)90129-Z. PubMed: 1555239. 1555239 10.1016/0092-8674(92)90129-z

[4] van de Wetering M, Clevers H (1992) Sequence-specific interaction of the HMG box proteins TCF-1 and SRY occurs within the minor groove of a Watson-Crick double helix. EMBO J 11: 3039-3044. PubMed: 1639073. 1639073 10.1002/j.1460-2075.1992.tb05374.x PMC556786

[5] Travis A, Amsterdam A, Belanger C, Grosschedl R (1991) LEF-1, a gene encoding a lymphoid-specific protein with an HMG domain, regulates T-cell receptor alpha enhancer function [corrected]. Genes Dev 5: 880-894. doi:10.1101/gad.5.5.880. PubMed: 1827423. 1827423 10.1101/gad.5.5.880

[6] van de Wetering M, Oosterwegel M, Dooijes D, Clevers H (1991) Identification and cloning of TCF-1, a T lymphocyte-specific transcription factor containing a sequence-specific HMG box. EMBO J 10: 123-132. PubMed: 1989880. 1989880 10.1002/j.1460-2075.1991.tb07928.x PMC452620

[7] Waterman ML, Fischer WH, Jones KA (1991) A thymus-specific member of the HMG protein family regulates the human T cell receptor C alpha enhancer. Genes Dev 5: 656-669. doi:10.1101/gad.5.4.656. PubMed: 2010090. 2010090 10.1101/gad.5.4.656

[8] Galceran J, Fariñas I, Depew MJ, Clevers H, Grosschedl R (1999) Wnt3a-/--like phenotype and limb deficiency in Lef1(-/-)Tcf1(-/-) mice. Genes Dev 13: 709-717. doi:10.1101/gad.13.6.709. PubMed: 10090727. 10090727 10.1101/gad.13.6.709 PMC316557

[9] Galceran J, Miyashita-Lin EM, Devaney E, Rubenstein JL, Grosschedl R (2000) Hippocampus development and generation of dentate gyrus granule cells is regulated by LEF1. Development 127: 469-482. PubMed: 10631168. 10631168 10.1242/dev.127.3.469

[10] Kim CH, Oda T, Itoh M, Jiang D, Artinger KB et al. (2000) Repressor activity of Headless/Tcf3 is essential for vertebrate head formation. Nature 407: 913-916. doi:10.1038/35038097. PubMed: 11057671. 11057671 10.1038/35038097 PMC4018833

[11] Korinek V, Barker N, Willert K, Molenaar M, Roose J et al. (1998) Two members of the Tcf family implicated in Wnt/beta-catenin signaling during embryogenesis in the mouse. Mol Cell Biol 18: 1248-1256. PubMed: 9488439. 9488439 10.1128/mcb.18.3.1248 PMC108837

[12] Lin R, Thompson S, Priess JR (1995) pop-1 encodes an HMG box protein required for the specification of a mesoderm precursor in early C. elegans embryos. Cell 83: 599-609. doi:10.1016/0092-8674(95)90100-0. PubMed: 7585963. 7585963 10.1016/0092-8674(95)90100-0

[13] Molenaar M, van de Wetering M, Oosterwegel M, Peterson-Maduro J, Godsave S et al. (1996) XTcf-3 transcription factor mediates beta-catenin-induced axis formation in Xenopus embryos. Cell 86: 391-399. doi:10.1016/S0092-8674(00)80112-9. PubMed: 8756721. 8756721 10.1016/s0092-8674(00)80112-9

[14] Pelegri F, Maischein HM (1998) Function of zebrafish beta-catenin and TCF-3 in dorsoventral patterning. Mech Dev 77: 63-74. doi:10.1016/S0925-4773(98)00132-4. PubMed: 9784608. 9784608 10.1016/s0925-4773(98)00132-4

[15] van de Wetering M, Cavallo R, Dooijes D, van Beest M, van Es J et al. (1997) Armadillo coactivates transcription driven by the product of the Drosophila segment polarity gene dTCF. Cell 88: 789-799. doi:10.1016/S0092-8674(00)81925-X. PubMed: 9118222. 9118222 10.1016/s0092-8674(00)81925-x

[16] Barker N, Huls G, Korinek V, Clevers H (1999) Restricted high level expression of Tcf-4 protein in intestinal and mammary gland epithelium. Am J Pathol 154: 29-35. doi:10.1016/S0002-9440(10)65247-9. PubMed: 9916915. 9916915 10.1016/S0002-9440(10)65247-9 PMC1853446

[17] Korinek V, Barker N, Moerer P, van Donselaar E, Huls G et al. (1998) Depletion of epithelial stem-cell compartments in the small intestine of mice lacking Tcf-4. Nat Genet 19: 379-383. doi:10.1038/1270. PubMed: 9697701. 9697701 10.1038/1270

[18] Korinek V, Barker N, Morin PJ, van Wichen D, de Weger R et al. (1997) Constitutive transcriptional activation by a beta-catenin-Tcf complex in APC-/- colon carcinoma. Science 275: 1784-1787. PubMed: 9065401. 9065401 10.1126/science.275.5307.1784

[19] Muncan V, Faro A, Haramis AP, Hurlstone AF, Wienholds E et al. (2007) T-cell factor 4 (Tcf7l2) maintains proliferative compartments in zebrafish intestine. EMBO Rep 8: 966-973. doi:10.1038/sj.embor.7401071. PubMed: 17823612. 17823612 10.1038/sj.embor.7401071 PMC2002560

[20] Behrens J, von Kries JP, Kühl M, Bruhn L, Wedlich D et al. (1996) Functional interaction of beta-catenin with the transcription factor LEF-1. Nature 382: 638-642. doi:10.1038/382638a0. PubMed: 8757136. 8757136 10.1038/382638a0

[21] Huber O, Korn R, McLaughlin J, Ohsugi M, Herrmann BG et al. (1996) Nuclear localization of beta-catenin by interaction with transcription factor LEF-1. Mech Dev 59: 3-10. doi:10.1016/0925-4773(96)00597-7. PubMed: 8892228. 8892228 10.1016/0925-4773(96)00597-7

[22] Cadigan KM, Nusse R (1997) Wnt signaling: a common theme in animal development. Genes Dev 11: 3286-3305. doi:10.1101/gad.11.24.3286. PubMed: 9407023. 9407023 10.1101/gad.11.24.3286

[23] Huelsken J, Behrens J (2002) The Wnt signalling pathway. J Cell Sci 115: 3977-3978. doi:10.1242/jcs.00089. PubMed: 12356903. 12356903 10.1242/jcs.00089

[24] Logan CY, Nusse R (2004) The Wnt signaling pathway in development and disease. Annu Rev Cell Dev Biol 20: 781-810. PubMed: 15473860. 15473860 10.1146/annurev.cellbio.20.010403.113126

[25] Munemitsu S, Albert I, Souza B, Rubinfeld B, Polakis P (1995) Regulation of intracellular beta-catenin levels by the adenomatous polyposis coli (APC) tumor-suppressor protein. Proc Natl Acad Sci U S A 92: 3046-3050. doi:10.1073/pnas.92.7.3046. PubMed: 7708772. 7708772 10.1073/pnas.92.7.3046 PMC42356

[26] Rubinfeld B, Souza B, Albert I, Müller O, Chamberlain SH et al. (1993) Association of the APC gene product with beta-catenin. Science 262: 1731-1734. doi:10.1126/science.8259518. PubMed: 8259518. 8259518 10.1126/science.8259518

[27] Su LK, Vogelstein B, Kinzler KW (1993) Association of the APC tumor suppressor protein with catenins. Science 262: 1734-1737. doi:10.1126/science.8259519. PubMed: 8259519. 8259519 10.1126/science.8259519

[28] Morin PJ, Sparks AB, Korinek V, Barker N, Clevers H et al. (1997) Activation of beta-catenin-Tcf signaling in colon cancer by mutations in beta-catenin or APC. Science 275: 1787-1790. PubMed: 9065402. 9065402 10.1126/science.275.5307.1787

[29] van de Wetering M, Sancho E, Verweij C, de Lau W, Oving I et al. (2002) The beta-catenin/TCF-4 complex imposes a crypt progenitor phenotype on colorectal cancer cells. Cell 111: 241-250. doi:10.1016/S0092-8674(02)01014-0. PubMed: 12408868. 12408868 10.1016/s0092-8674(02)01014-0

[30] Behrens J, Jerchow BA, Würtele M, Grimm J, Asbrand C et al. (1998) Functional interaction of an axin homolog, conductin, with beta-catenin, APC, and GSK3beta. Science 280: 596-599. PubMed: 9554852. 9554852 10.1126/science.280.5363.596

[31] Hart MJ, de los Santos R, Albert IN, Rubinfeld B, Polakis P (1998) Downregulation of beta-catenin by human Axin and its association with the APC tumor suppressor, beta-catenin and GSK3 beta. Curr Biol 8: 573-581. doi:10.1016/S0960-9822(98)70226-X. PubMed: 9601641. 9601641 10.1016/s0960-9822(98)70226-x

[32] Ikeda S, Kishida S, Yamamoto H, Murai H, Koyama S et al. (1998) Axin, a negative regulator of the Wnt signaling pathway, forms a complex with GSK-3beta and beta-catenin and promotes GSK-3beta-dependent phosphorylation of beta-catenin. EMBO J 17: 1371-1384. PubMed: 9482734. 9482734 10.1093/emboj/17.5.1371 PMC1170485

[33] Rubinfeld B, Albert I, Porfiri E, Fiol C, Munemitsu S et al. (1996) Binding of GSK3beta to the APC-beta-catenin complex and regulation of complex assembly. Science 272: 1023-1026. doi:10.1126/science.272.5264.1023. PubMed: 8638126. 8638126 10.1126/science.272.5264.1023

[34] Sakanaka C, Weiss JB, Williams LT (1998) Bridging of beta-catenin and glycogen synthase kinase-3beta by axin and inhibition of beta-catenin-mediated transcription. Proc Natl Acad Sci U S A 95: 3020-3023. PubMed: 9501208. 9501208 10.1073/pnas.95.6.3020 PMC19687

[35] Yost C, Torres M, Miller JR, Huang E, Kimelman D et al. (1996) The axis-inducing activity, stability, and subcellular distribution of beta-catenin is regulated in Xenopus embryos by glycogen synthase kinase 3. Genes Dev 10: 1443-1454. PubMed: 8666229. 8666229 10.1101/gad.10.12.1443

[36] Zeng L, Fagotto F, Zhang T, Hsu W, Vasicek TJ et al. (1997) The mouse Fused locus encodes Axin, an inhibitor of the Wnt signaling pathway that regulates embryonic axis formation. Cell 90: 181-192. doi:10.1016/S0092-8674(00)80324-4. PubMed: 9230313. 9230313 10.1016/s0092-8674(00)80324-4

[37] Peifer M, Pai LM, Casey M (1994) Phosphorylation of the Drosophila adherens junction protein Armadillo: roles for wingless signal and zeste-white 3 kinase. Dev Biol 166: 543-556. doi:10.1006/dbio.1994.1336. PubMed: 7529201. 7529201 10.1006/dbio.1994.1336

[38] Peifer M, Sweeton D, Casey M, Wieschaus E (1994) wingless signal and Zeste-white 3 kinase trigger opposing changes in the intracellular distribution of Armadillo. Development 120: 369-380. PubMed: 8149915. 8149915 10.1242/dev.120.2.369

[39] Siegfried E, Chou TB, Perrimon N (1992) wingless signaling acts through zeste-white 3, the Drosophila homolog of glycogen synthase kinase-3, to regulate engrailed and establish cell fate. Cell 71: 1167-1179. PubMed: 1335365. 1335365 10.1016/s0092-8674(05)80065-0

[40] Siegfried E, Wilder EL, Perrimon N (1994) Components of wingless signalling in Drosophila. Nature 367: 76-80. PubMed: 8107779. 8107779 10.1038/367076a0

[41] Aberle H, Bauer A, Stappert J, Kispert A, Kemler R (1997) beta-catenin is a target for the ubiquitin-proteasome pathway. EMBO J 16: 3797-3804. PubMed: 9233789. 9233789 10.1093/emboj/16.13.3797 PMC1170003

[42] Kimelman D, Xu W (2006) beta-catenin destruction complex: insights and questions from a structural perspective. Oncogene 25: 7482-7491. PubMed: 17143292. 17143292 10.1038/sj.onc.1210055

[43] Brannon M, Gomperts M, Sumoy L, Moon RT, Kimelman D (1997) A beta-catenin/XTcf-3 complex binds to the siamois promoter to regulate dorsal axis specification in Xenopus. Genes Dev 11: 2359-2370. PubMed: 9308964. 9308964 10.1101/gad.11.18.2359 PMC316518

[44] Riese J, Yu X, Munnerlyn A, Eresh S, Hsu SC et al. (1997) LEF-1, a nuclear factor coordinating signaling inputs from wingless and decapentaplegic. Cell 88: 777-787. doi:10.1016/S0092-8674(00)81924-8. PubMed: 9118221. 9118221 10.1016/s0092-8674(00)81924-8

[45] Rocheleau CE, Downs WD, Lin R, Wittmann C, Bei Y et al. (1997) Wnt signaling and an APC-related gene specify endoderm in early C. elegans embryos. Cell 90: 707-716. PubMed: 9288750. 9288750 10.1016/s0092-8674(00)80531-0

[46] Thorpe CJ, Schlesinger A, Carter JC, Bowerman B (1997) Wnt signaling polarizes an early C. elegans blastomere to distinguish endoderm from mesoderm. Cell 90: 695-705. PubMed: 9288749. 9288749 10.1016/s0092-8674(00)80530-9

[47] Cavallo RA, Cox RT, Moline MM, Roose J, Polevoy GA et al. (1998) Drosophila Tcf and Groucho interact to repress Wingless signalling activity. Nature 395: 604-608. PubMed: 9783586. 9783586 10.1038/26982

[48] Levanon D, Goldstein RE, Bernstein Y, Tang H, Goldenberg D et al. (1998) Transcriptional repression by AML1 and LEF-1 is mediated by the TLE/Groucho corepressors. Proc Natl Acad Sci U S A 95: 11590-11595. PubMed: 9751710. 9751710 10.1073/pnas.95.20.11590 PMC21685

[49] Roose J, Molenaar M, Peterson J, Hurenkamp J, Brantjes H et al. (1998) The Xenopus Wnt effector XTcf-3 interacts with Groucho-related transcriptional repressors. Nature 395: 608-612. PubMed: 9783587. 9783587 10.1038/26989

[50] Merrill BJ, Pasolli HA, Polak L, Rendl M, García-García MJ et al. (2004) Tcf3: a transcriptional regulator of axis induction in the early embryo. Development 131: 263-274. PubMed: 14668413. 14668413 10.1242/dev.00935

[51] Tsuji S, Hashimoto C (2005) Choice of either beta-catenin or Groucho/TLE as a co-factor for Xtcf-3 determines dorsal-ventral cell fate of diencephalon during Xenopus development. Dev Genes Evol 215: 275-284. PubMed: 15747128. 15747128 10.1007/s00427-005-0474-0

[52] Guger KA, Gumbiner BM (1995) beta-Catenin has Wnt-like activity and mimics the Nieuwkoop signaling center in Xenopus dorsal-ventral patterning. Dev Biol 172: 115-125. PubMed: 7589792. 7589792 10.1006/dbio.1995.0009

[53] Holland LZ (2002) Heads or tails? Amphioxus and the evolution of anterior-posterior patterning in deuterostomes. Dev Biol 241: 209-228. PubMed: 11784106. 11784106 10.1006/dbio.2001.0503

[54] Kelly GM, Erezyilmaz DF, Moon RT (1995) Induction of a secondary embryonic axis in zebrafish occurs following the overexpression of beta-catenin. Mech Dev 53: 261-273. PubMed: 8562427. 8562427 10.1016/0925-4773(95)00442-4

[55] Larabell CA, Torres M, Rowning BA, Yost C, Miller JR et al. (1997) Establishment of the dorso-ventral axis in Xenopus embryos is presaged by early asymmetries in beta-catenin that are modulated by the Wnt signaling pathway. J Cell Biol 136: 1123-1136. PubMed: 9060476. 9060476 10.1083/jcb.136.5.1123 PMC2132470

[56] Schneider S, Steinbeisser H, Warga RM, Hausen P (1996) Beta-catenin translocation into nuclei demarcates the dorsalizing centers in frog and fish embryos. Mech Dev 57: 191-198. PubMed: 8843396. 8843396 10.1016/0925-4773(96)00546-1

[57] Sumoy L, Kiefer J, Kimelman D (1999) Conservation of intracellular Wnt signaling components in dorsal-ventral axis formation in zebrafish. Dev Genes Evol 209: 48-58. PubMed: 9914418. 9914418 10.1007/s004270050226

[58] Vonica A, Weng W, Gumbiner BM, Venuti JM (2000) TCF is the nuclear effector of the beta-catenin signal that patterns the sea urchin animal-vegetal axis. Dev Biol 217: 230-243. PubMed: 10625549. 10625549 10.1006/dbio.1999.9551

[59] Fisher AL, Caudy M (1998) Groucho proteins: transcriptional corepressors for specific subsets of DNA-binding transcription factors in vertebrates and invertebrates. Genes Dev 12: 1931-1940. PubMed: 9649497. 9649497 10.1101/gad.12.13.1931

[60] Giebel B, Campos-Ortega JA (1997) Functional dissection of the Drosophila enhancer of split protein, a suppressor of neurogenesis. Proc Natl Acad Sci U S A 94: 6250-6254. PubMed: 9177203. 9177203 10.1073/pnas.94.12.6250 PMC21035

[61] De Iaco R, Soustelle L, Kammerer M, Sorrentino S, Jacques C et al. (2006) Huckebein-mediated autoregulation of Glide/Gcm triggers glia specification. EMBO J 25: 244-254. PubMed: 16362045. 16362045 10.1038/sj.emboj.7600907 PMC1356350

[62] Brantjes H, Roose J, van de Wetering M, Clevers H (2001) All Tcf HMG box transcription factors interact with Groucho-related co-repressors. Nucleic Acids Res 29: 1410-1419. PubMed: 11266540. 11266540 10.1093/nar/29.7.1410 PMC31284

[63] Lepourcelet M, Shivdasani RA (2002) Characterization of a novel mammalian Groucho isoform and its role in transcriptional regulation. J Biol Chem 277: 47732-47740. PubMed: 12359720. 12359720 10.1074/jbc.M208154200

[64] Range RC, Venuti JM, McClay DR (2005) LvGroucho and nuclear beta-catenin functionally compete for Tcf binding to influence activation of the endomesoderm gene regulatory network in the sea urchin embryo. Dev Biol 279: 252-267. PubMed: 15708573. 15708573 10.1016/j.ydbio.2004.12.023

[65] Han K, Manley JL (1993) Transcriptional repression by the Drosophila even-skipped protein: definition of a minimal repression domain. Genes Dev 7: 491-503. PubMed: 8095483. 8095483 10.1101/gad.7.3.491

[66] Yu X, Li P, Roeder RG, Wang Z (2001) Inhibition of androgen receptor-mediated transcription by amino-terminal enhancer of split. Mol Cell Biol 21: 4614-4625. PubMed: 11416139. 11416139 10.1128/MCB.21.14.4614-4625.2001 PMC87125

[67] Ren B, Chee KJ, Kim TH, Maniatis T (1999) PRDI-BF1/Blimp-1 repression is mediated by corepressors of the Groucho family of proteins. Genes Dev 13: 125-137. PubMed: 9887105. 9887105 10.1101/gad.13.1.125 PMC316372

[68] Tetsuka T, Uranishi H, Imai H, Ono T, Sonta S et al. (2000) Inhibition of nuclear factor-kappaB-mediated transcription by association with the amino-terminal enhancer of split, a Groucho-related protein lacking WD40 repeats. J Biol Chem 275: 4383-4390. PubMed: 10660609. 10660609 10.1074/jbc.275.6.4383

[69] Arce L, Pate KT, Waterman ML (2009) Groucho binds two conserved regions of LEF-1 for HDAC-dependent repression. BMC Cancer 9: 159 PubMed: 19460168. 19460168 10.1186/1471-2407-9-159 PMC2701438

[70] Rundlett SE, Carmen AA, Suka N, Turner BM, Grunstein M (1998) Transcriptional repression by UME6 involves deacetylation of lysine 5 of histone H4 by RPD3. Nature 392: 831-835. PubMed: 9572144. 9572144 10.1038/33952

[71] Sarma NJ, Yaseen NR (2011) Amino-terminal enhancer of split (AES) interacts with the oncoprotein NUP98-HOXA9 and enhances its transforming ability. J Biol Chem 286: 38989-39001. PubMed: 21937451. 21937451 10.1074/jbc.M111.297952 PMC3234724

[72] Gietz D, St Jean A, Woods RA, Schiestl RH (1992) Improved method for high efficiency transformation of intact yeast cells. Nucleic Acids Res 20: 1425 PubMed: 1561104. 1561104 10.1093/nar/20.6.1425 PMC312198

[73] Akimenko MA, Johnson SL, Westerfield M, Ekker M (1995) Differential induction of four msx homeobox genes during fin development and regeneration in zebrafish. Development 121: 347-357. PubMed: 7768177. 7768177 10.1242/dev.121.2.347

[74] Fisher S, Grice EA, Vinton RM, Bessling SL, Urasaki A et al. (2006) Evaluating the biological relevance of putative enhancers using Tol2 transposon-mediated transgenesis in zebrafish. Nat Protoc 1: 1297-1305. doi:10.1038/nprot.2006.230. PubMed: 17406414. 17406414 10.1038/nprot.2006.230

[75] Miller-Bertoglio VE, Fisher S, Sánchez A, Mullins MC, Halpern ME (1997) Differential regulation of chordin expression domains in mutant zebrafish. Dev Biol 192: 537-550. doi:10.1006/dbio.1997.8788. PubMed: 9441687. 9441687 10.1006/dbio.1997.8788

[76] Detrich HW 3rd, Kieran MW, Chan FY, Barone LM, Yee K et al. (1995) Intraembryonic hematopoietic cell migration during vertebrate development. Proc Natl Acad Sci U S A 92: 10713-10717. doi:10.1073/pnas.92.23.10713. PubMed: 7479870. 7479870 10.1073/pnas.92.23.10713 PMC40682

[77] Zhao J, Cao Y, Zhao C, Postlethwait J, Meng A (2003) An SP1-like transcription factor Spr2 acts downstream of Fgf signaling to mediate mesoderm induction. EMBO J 22: 6078-6088. PubMed: 14609954. 14609954 10.1093/emboj/cdg593 PMC275448

[78] Thisse C, Thisse B (2008) High-resolution in situ hybridization to whole-mount zebrafish embryos. Nat Protoc 3: 59-69. doi:10.1038/nnano.2008.25. PubMed: 18193022. 18193022 10.1038/nprot.2007.514

[79] Weise A, Bruser K, Elfert S, Wallmen B, Wittel Y et al. (2010) Alternative splicing of Tcf7l2 transcripts generates protein variants with differential promoter-binding and transcriptional activation properties at Wnt/beta-catenin targets. Nucleic Acids Res 38: 1964-1981. doi:10.1093/nar/gkp1197. PubMed: 20044351. 20044351 10.1093/nar/gkp1197 PMC2847248

[80] Valenta T, Lukas J, Korinek V (2003) HMG box transcription factor TCF-4’s interaction with CtBP1 controls the expression of the Wnt target Axin2/Conduction in human embryonic kidney cells. Nucleic Acids Res 31: 2369-2380. PubMed: 12711682. 12711682 10.1093/nar/gkg346 PMC154232

[81] Pukrop T, Gradl D, Henningfeld KA, Knochel W, Wedlich D et al. (2001) Identification of two regulatory elements within the high mobility group box transcription factor XTCF-4. J Biol Chem 276: 8968-8978. PubMed: 11124256. 11124256 10.1074/jbc.M007533200

[82] Arce L, Yokoyama NN, Waterman ML (2006) Diversity of LEF/TCF action in development and disease. Oncogene 25: 7492-7504. PubMed: 17143293. 17143293 10.1038/sj.onc.1210056

[83] Brannon M, Brown JD, Bates R, Kimelman D, Moon RT (1999) XCtBP is a XTcf-3 co-repressor with roles throughout Xenopus development. Development 126: 3159-3170. PubMed: 10375506. 10375506 10.1242/dev.126.14.3159

[84] Goldstein RE, Jiménez G, Cook O, Gur D, Paroush Z (1999) Huckebein repressor activity in Drosophila terminal patterning is mediated by Groucho. Development 126: 3747-3755. PubMed: 10433905. 10433905 10.1242/dev.126.17.3747

[85] Gradl D, König A, Wedlich D (2002) Functional diversity of Xenopus lymphoid enhancer factor/T-cell factor transcription factors relies on combinations of activating and repressing elements. J Biol Chem 277: 14159-14171. PubMed: 11821382. 11821382 10.1074/jbc.M107055200

[86] Beagle B, Johnson GV (2010) AES/GRG5: more than just a dominant-negative TLE/GRG family member. Dev Dyn 239: 2795-2805. PubMed: 20925119. 20925119 10.1002/dvdy.22439 PMC2997355

[87] Fisher AL, Ohsako S, Caudy M (1996) The WRPW motif of the hairy-related basic helix-loop-helix repressor proteins acts as a 4-amino-acid transcription repression and protein–protein interaction domain. Mol Cell Biol 16: 2670-2677. PubMed: 8649374. 8649374 10.1128/mcb.16.6.2670 PMC231257

[88] Schier AF, Talbot WS (2005) Molecular genetics of axis formation in zebrafish. Annu Rev Genet 39: 561-613. PubMed: 16285872. 16285872 10.1146/annurev.genet.37.110801.143752

[89] Kelly C, Chin AJ, Leatherman JL, Kozlowski DJ, Weinberg ES (2000) Maternally controlled (beta)-catenin-mediated signaling is required for organizer formation in the zebrafish. Development 127: 3899-3911. PubMed: 10952888. 10952888 10.1242/dev.127.18.3899

[90] McMahon AP, Moon RT (1989) Ectopic expression of the proto-oncogene int-1 in Xenopus embryos leads to duplication of the embryonic axis. Cell 58: 1075-1084. PubMed: 2673541. 2673541 10.1016/0092-8674(89)90506-0

[91] Funayama N, Fagotto F, McCrea P, Gumbiner BM (1995) Embryonic axis induction by the armadillo repeat domain of beta-catenin: evidence for intracellular signaling. J Cell Biol 128: 959-968. doi:10.1083/jcb.128.5.959. PubMed: 7876319. 7876319 10.1083/jcb.128.5.959 PMC2120405

[92] McCrea PD, Brieher WM, Gumbiner BM (1993) Induction of a secondary body axis in Xenopus by antibodies to beta-catenin. J Cell Biol 123: 477-484. doi:10.1083/jcb.123.2.477. PubMed: 8408227. 8408227 10.1083/jcb.123.2.477 PMC2119835

[93] Weidinger G, Thorpe CJ, Wuennenberg-Stapleton K, Ngai J, Moon RT (2005) The Sp1-related transcription factors sp5 and sp5-like act downstream of Wnt/beta-catenin signaling in mesoderm and neuroectoderm patterning. Curr Biol 15: 489-500. doi:10.1016/j.cub.2005.01.041. PubMed: 15797017. 15797017 10.1016/j.cub.2005.01.041

[94] Thorpe CJ, Weidinger G, Moon RT (2005) Wnt/beta-catenin regulation of the Sp1-related transcription factor sp5l promotes tail development in zebrafish. Development 132: 1763-1772. doi:10.1242/dev.01733. PubMed: 15772132. 15772132 10.1242/dev.01733

[95] Wang H, Lei Q, Oosterveen T, Ericson J, Matise MP (2011) Tcf/Lef repressors differentially regulate Shh-Gli target gene activation thresholds to generate progenitor patterning in the developing CNS. Development 138: 3711-3721. doi:10.1242/dev.068270. PubMed: 21775418. 21775418 10.1242/dev.068270 PMC3152926

[96] Kunz M, Herrmann M, Wedlich D, Gradl D (2004) Autoregulation of canonical Wnt signaling controls midbrain development. Dev Biol 273: 390-401. doi:10.1016/j.ydbio.2004.06.015. PubMed: 15328021. 15328021 10.1016/j.ydbio.2004.06.015

[97] Nguyen H, Merrill BJ, Polak L, Nikolova M, Rendl M et al. (2009) Tcf3 and Tcf4 are essential for long-term homeostasis of skin epithelia. Nat Genet 41: 1068-1075. doi:10.1038/ng.431. PubMed: 19718027. 19718027 10.1038/ng.431 PMC2792754

[98] Mariadason JM, Bordonaro M, Aslam F, Shi L, Kuraguchi M et al. (2001) Down-regulation of beta-catenin TCF signaling is linked to colonic epithelial cell differentiation. Cancer Res 61: 3465-3471. PubMed: 11309309. 11309309

[99] Struewing I, Boyechko T, Barnett C, Beildeck M, Byers SW et al. (2010) The balance of TCF7L2 variants with differential activities in Wnt-signaling is regulated by lithium in a GSK3beta-independent manner. Biochem Biophys Res Commun 399: 245-250. PubMed: 20654575. 20654575 10.1016/j.bbrc.2010.07.062 PMC2926262

[100] Atcha FA, Syed A, Wu B, Hoverter NP, Yokoyama NN et al. (2007) A unique DNA binding domain converts T-cell factors into strong Wnt effectors. Mol Cell Biol 27: 8352-8363. PubMed: 17893322. 17893322 10.1128/MCB.02132-06 PMC2169181

[101] Duval A, Rolland S, Tubacher E, Bui H, Thomas G et al. (2000) The human T-cell transcription factor-4 gene: structure, extensive characterization of alternative splicings, and mutational analysis in colorectal cancer cell lines. Cancer Res 60: 3872-3879. PubMed: 10919662. 10919662

[102] Brinkmeier ML, Potok MA, Cha KB, Gridley T, Stifani S et al. (2003) TCF and Groucho-related genes influence pituitary growth and development. Mol Endocrinol 17: 2152-2161. PubMed: 12907761. 12907761 10.1210/me.2003-0225

[103] Douglas KR, Brinkmeier ML, Kennell JA, Eswara P, Harrison TA et al. (2001) Identification of members of the Wnt signaling pathway in the embryonic pituitary gland. Mamm Genome 12: 843-851. doi:10.1007/s00335-001-2076-0. PubMed: 11845287. 11845287 10.1007/s00335-001-2076-0

[104] Young RM, Reyes AE, Allende ML (2002) Expression and splice variant analysis of the zebrafish tcf4 transcription factor. Mech Dev 117: 269-273. PubMed: 12204269. 12204269 10.1016/s0925-4773(02)00180-6

[105] Cho EA, Dressler GR (1998) TCF-4 binds beta-catenin and is expressed in distinct regions of the embryonic brain and limbs. Mech Dev 77: 9-18. doi:10.1016/S0925-4773(98)00131-2. PubMed: 9784592. 9784592 10.1016/s0925-4773(98)00131-2

[106] Giese K, Grosschedl R (1993) LEF-1 contains an activation domain that stimulates transcription only in a specific context of factor-binding sites. EMBO J 12: 4667-4676. PubMed: 8223476. 8223476 10.1002/j.1460-2075.1993.tb06155.x PMC413904

[107] Carlsson P, Waterman ML, Jones KA (1993) The hLEF/TCF-1 alpha HMG protein contains a context-dependent transcriptional activation domain that induces the TCR alpha enhancer in T cells. Genes Dev 7: 2418-2430. doi:10.1101/gad.7.12a.2418. PubMed: 8253387. 8253387 10.1101/gad.7.12a.2418

[108] Gasperowicz M, Otto F (2005) Mammalian Groucho homologs: redundancy or specificity? J Cell Biochem 95: 670-687. doi:10.1002/jcb.20476. PubMed: 15861397. 15861397 10.1002/jcb.20476

[109] Wainwright SM, Ish-Horowicz D (1992) Point mutations in the Drosophila hairy gene demonstrate in vivo requirements for basic, helix-loop-helix, and WRPW domains. Mol Cell Biol 12: 2475-2483. PubMed: 1588951. 1588951 10.1128/mcb.12.6.2475-2483.1992 PMC364440

[110] Aronson BD, Fisher AL, Blechman K, Caudy M, Gergen JP (1997) Groucho-dependent and -independent repression activities of Runt domain proteins. Mol Cell Biol 17: 5581-5587. PubMed: 9271433. 9271433 10.1128/mcb.17.9.5581 PMC232406

[111] Um M, Li C, Manley JL (1995) The transcriptional repressor even-skipped interacts directly with TATA-binding protein. Mol Cell Biol 15: 5007-5016. PubMed: 7651419. 7651419 10.1128/mcb.15.9.5007 PMC230748

[112] Wang ZY, Qiu QQ, Gurrieri M, Huang J, Deuel TF (1995) WT1, the Wilms’ tumor suppressor gene product, represses transcription through an interactive nuclear protein. Oncogene 10: 1243-1247. PubMed: 7700651. 7700651

[113] Hanna-Rose W, Hansen U (1996) Active repression mechanisms of eukaryotic transcription repressors. Trends Genet 12: 229-234. PubMed: 8928228. 8928228 10.1016/0168-9525(96)10022-6

[114] Jennings BH, Ish-Horowicz D (2008) The Groucho/TLE/Grg family of transcriptional co-repressors. Genome Biol 9: 205. doi:10.1186/gb-2008-9-1-205. PubMed: 18254933. 18254933 10.1186/gb-2008-9-1-205 PMC2395242

[115] Sekiya T, Zaret KS (2007) Repression by Groucho/TLE/Grg proteins: genomic site recruitment generates compacted chromatin in vitro and impairs activator binding in vivo. Mol Cell 28: 291-303. doi:10.1016/j.molcel.2007.10.002. PubMed: 17964267. 17964267 10.1016/j.molcel.2007.10.002 PMC2083644

[116] Song H, Hasson P, Paroush Z, Courey AJ (2004) Groucho oligomerization is required for repression in vivo. Mol Cell Biol 24: 4341-4350. doi:10.1128/MCB.24.10.4341-4350.2004. PubMed: 15121853. 15121853 10.1128/MCB.24.10.4341-4350.2004 PMC400465

[117] Chen G, Nguyen PH, Courey AJ (1998) A role for Groucho tetramerization in transcriptional repression. Mol Cell Biol 18: 7259-7268. PubMed: 9819412. 9819412 10.1128/mcb.18.12.7259 PMC109307

[118] Pinto M, Lobe CG (1996) Products of the grg (Groucho-related gene) family can dimerize through the amino-terminal Q domain. J Biol Chem 271: 33026-33031. doi:10.1074/jbc.271.51.33026. PubMed: 8955148. 8955148 10.1074/jbc.271.51.33026

[119] Chen G, Courey AJ (2000) Groucho/TLE family proteins and transcriptional repression. Gene 249: 1-16. PubMed: 10831834. 10831834 10.1016/s0378-1119(00)00161-x

[120] Chen G, Fernandez J, Mische S, Courey AJ (1999) A functional interaction between the histone deacetylase Rpd3 and the corepressor groucho in Drosophila development. Genes Dev 13: 2218-2230. doi:10.1101/gad.13.17.2218. PubMed: 10485845. 10485845 10.1101/gad.13.17.2218 PMC316998

[121] Zhang Y, Gao S, Wang Z (2010) Structural and functional analysis of amino-terminal enhancer of split in androgen-receptor-driven transcription. Biochem J 427: 499-511. PubMed: 20163360. 20163360 10.1042/BJ20091631

[122] Flowers EB, Poole RJ, Tursun B, Bashllari E, Pe’er I et al. (2010) The Groucho ortholog UNC-37 interacts with the short Groucho-like protein LSY-22 to control developmental decisions in C. elegans. Development 137: 1799-1805. PubMed: 20431118. 20431118 10.1242/dev.046219 PMC2867316

[123] Daniels DL, Weis WI (2005) Beta-catenin directly displaces Groucho/TLE repressors from Tcf/Lef in Wnt-mediated transcription activation. Nat Struct Mol Biol 12: 364-371. PubMed: 15768032. 15768032 10.1038/nsmb912

[124] Beagle B, Johnson GV (2010) Differential modulation of TCF/LEF-1 activity by the soluble LRP6-ICD. PLOS ONE 5: e11821. doi:10.1371/journal.pone.0011821. PubMed: 20676368. 20676368 10.1371/journal.pone.0011821 PMC2911377

[125] Roose J, Huls G, van Beest M, Moerer P, van der Horn K et al. (1999) Synergy between tumor suppressor APC and the beta-catenin-Tcf4 target Tcf1. Science 285: 1923-1926. doi:10.1126/science.285.5435.1923. PubMed: 10489374. 10489374 10.1126/science.285.5435.1923

[126] Jennings BH, Wainwright SM, Ish-Horowicz D (2008) Differential in vivo requirements for oligomerization during Groucho-mediated repression. EMBO Rep 9: 76-83. doi:10.1038/sj.embor.7401122. PubMed: 18034187. 18034187 10.1038/sj.embor.7401122 PMC2246631

[127] Andrioli LP, Digiampietri LA, de Barros LP, Machado-Lima A (2012) Huckebein is part of a combinatorial repression code in the anterior blastoderm. Dev Biol 361: 177-185. doi:10.1016/j.ydbio.2011.10.016. PubMed: 22027434. 22027434 10.1016/j.ydbio.2011.10.016

[128] Dorsky RI, Snyder A, Cretekos CJ, Grunwald DJ, Geisler R et al. (1999) Maternal and embryonic expression of zebrafish lef1. Mech Dev 86: 147-150. PubMed: 10446273. 10446273 10.1016/s0925-4773(99)00101-x

[129] Yang J, Tan C, Darken RS, Wilson PA, Klein PS (2002) Beta-catenin/Tcf-regulated transcription prior to the midblastula transition. Development 129: 5743-5752. doi:10.1242/dev.00150. PubMed: 12421713. 12421713 10.1242/dev.00150

[130] Verkade H, Heath JK (2008) Wnt signaling mediates diverse developmental processes in zebrafish. Methods Mol Biol 469: 225-251. doi:10.1007/978-1-60327-469-2_17. PubMed: 19109714. 19109714 10.1007/978-1-60327-469-2_17

[131] Ashyraliyev M, Siggens K, Janssens H, Blom J, Akam M et al. (2009) Gene circuit analysis of the terminal gap gene huckebein. PLOS Comput Biol 5: e1000548. 19876378 10.1371/journal.pcbi.1000548 PMC2760955

[132] Shimeld SM (2008) C2H2 zinc finger genes of the Gli, Zic, KLF, SP, Wilms' tumour, Huckebein, Snail, Ovo, Spalt, Odd, Blimp-1, Fez and related gene families from Branchiostoma floridae. Dev Genes Evol 218: 639-649. 18795322 10.1007/s00427-008-0248-6

[133] Molenaar M, Brian E, Roose J, Clevers H, Destrée O (2000) Differential expression of the Groucho-related genes 4 and 5 during early development of Xenopus laevis. Mech Dev 91: 311-315. doi:10.1016/S0925-4773(99)00259-2. PubMed: 10704855. 10704855 10.1016/s0925-4773(99)00259-2

[134] Wang W, Wang YG, Reginato AM, Glotzer DJ, Fukai N et al. (2004) Groucho homologue Grg5 interacts with the transcription factor Runx2-Cbfa1 and modulates its activity during postnatal growth in mice. Dev Biol 270: 364-381. PubMed: 15183720. 15183720 10.1016/j.ydbio.2004.03.003

[135] Standley HJ, Destree O, Kofron M, Wylie C, Heasman J (2006) Maternal XTcf1 and XTcf4 have distinct roles in regulating Wnt target genes. Dev Biol 289: 318-328. PubMed: 16325796. 16325796 10.1016/j.ydbio.2005.10.012

[136] Liu F, van den Broek O, Destree O, Hoppler S (2005) Distinct roles for Xenopus Tcf/Lef genes in mediating specific responses to Wnt/beta-catenin signalling in mesoderm development. Development 132: 5375-5385. doi:10.1242/dev.02152. PubMed: 16291789. 16291789 10.1242/dev.02152

