# Supplementary figures and images for: GRG5/AES Interacts with T-Cell Factor 4 (TCF4) and Downregulates Wnt Signaling in Human Cells and Zebrafish Embryos
Source: PLoS One. 2013 Jul 1;8(7):e67694. doi: 10.1371/journal.pone.0067694 (PMC3698143; doi:10.1371/journal.pone.0067694)

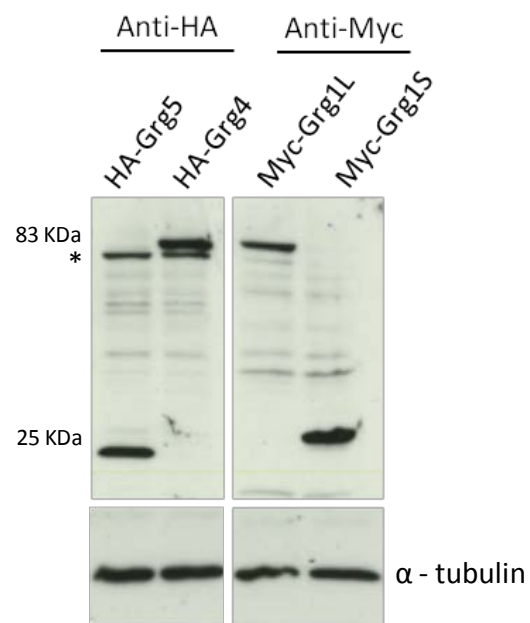

**Figure S1**

Supplement: Figure S1 — All Grgs tested were highly expressed in HEK293 cells. HEK293 cells were transiently transfected with the Grg expression plasmids indicated above the lines, and 25 μg of total protein was loaded for Western-blot analysis, using either anti-HA or anti-Myc antibodies. All transfections resulted in high levels of expression for the corresponding proteins, but quantitative comparisons are limited by the use of different epitopes. The asterisk indicates a nonspecific band around 80 KDa, detected by the anti-HA antibody. Membranes were reprobed with an antibody specific for α - tubulin, which served as a loading control (lower blot). (PDF) [file pone.0067694.s001.pdf]

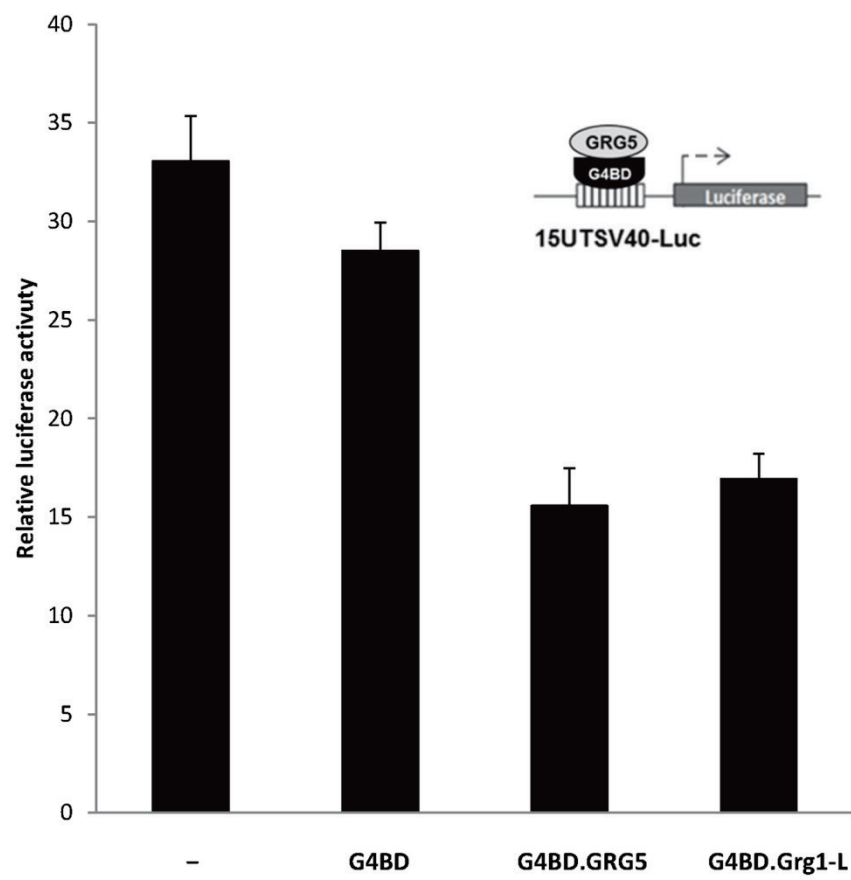

Figure S2

Supplement: Figure S2 — GRG5/AES has intrinsic repressive activity when directly bound to DNA. HEK293 cells were transfected with the 15UTSV-Luc reporter alone or together with the indicated effector plasmids. 15UTSV-Luc has high basal activity (resulting from the presence of an SV40 promoter) and is Gal4 responsive (because it contains 15 UASG). Reporter activity repression by a Gal4-binding domain-GRG5/AES fusion (G4BD.GRG5) is similar to that of its Gal4-Grg1-L counterpart. An expression vector encoding only the Gal4-binding domain (G4BD) was used as a negative control. (PDF) [file pone.0067694.s002.pdf]

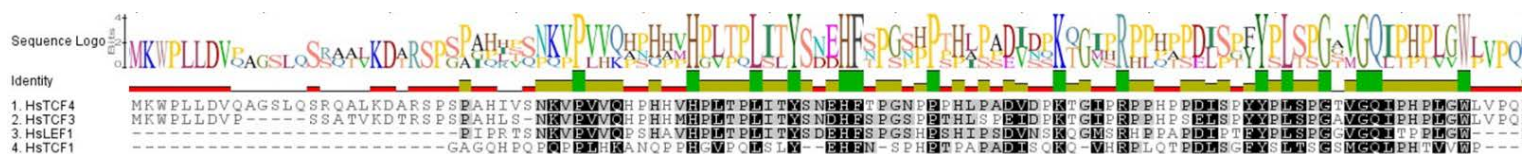

Figure S4

Supplement: Figure S4 — GRG-binding regions in TCF/LEFs display high proline content. Alignment of the 111-amino acid (amino acids 130-240) human TCF4 stretch to which we mapped GRG5/AES-binding to the other TCF/LEF human protein sequences using the Clustal W algorithm running on the Geneious v 5.3 program. (PDF) [file pone.0067694.s004.pdf]
