# Supplementary material for: GRG5/AES Interacts with T-Cell Factor 4 (TCF4) and Downregulates Wnt Signaling in Human Cells and Zebrafish Embryos
Source: PLoS One. 2013 Jul 1;8(7):e67694. doi: 10.1371/journal.pone.0067694 (PMC3698143; doi:10.1371/journal.pone.0067694)
Supplement: Figure S3 — Amino acid alignment of human TCF/LEF family members. Amino acid numbers for each protein are indicated on the right. Black shading indicates identity, grey shading indicates similar residues. The two highest regions of homology are the N-terminal β-catenin binding domain (BD) (amino acids 1-53 of TCF4) and the HMG-box (amino acids 318-409 of TCF4). The GRG5/AES-interacting region in TCF4 mapped in this study (red box) corresponds to a 111 amino-acid stretch in the most variable region among TCFs (amino acids 130-240 of TCF4). The previously described [49] GRG/TLE-binding domain in TCF1 (amino acids 176-359 of TCF1) is delimited by asterisks (*). Blue boxes highlight three specific domains: LVPQ (amino acids 237-240 of TCF4) contained in the GRG5/AES-interacting region of TCF4; FRHPY (amino acids 253-257 of TCF4), not necessary for this interaction, and FPPHMV (amino acids 270-275 amino acids in TCF4), corresponding to the aligned region of a previously characterized domain involved in Groucho-binding for LEF1 [69], but not necessary for TCF4-GRG5/AES interaction. Sequences were aligned using the Clustal W algorithm. (PDF) [file pone.0067694.s003.pdf]

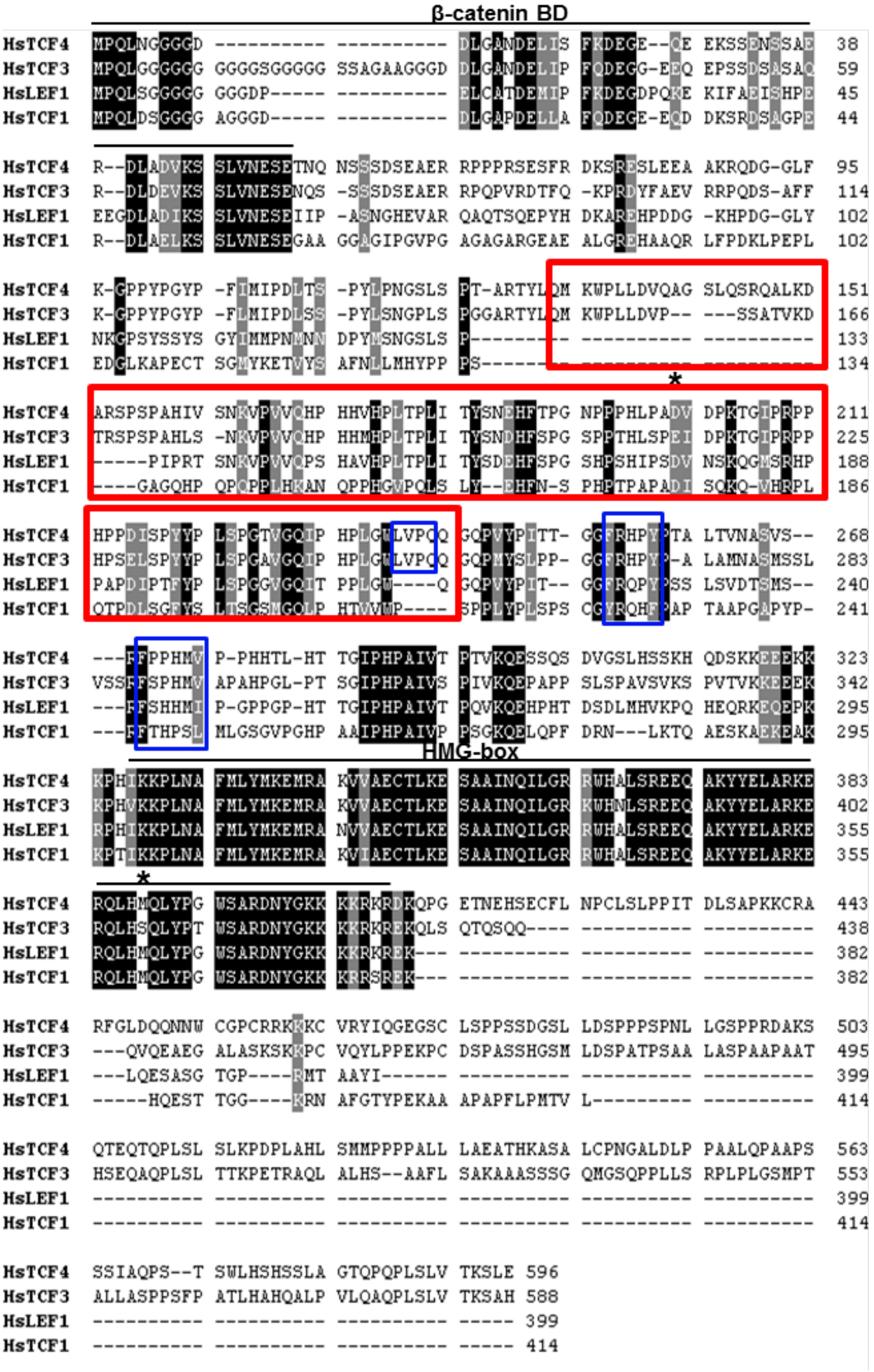
β-catenin BD
HsTCF4 MPQLNGGGGD ---------- ---------- DLGANDELIS FKDEGE--QE EKSSENSSAE 38
HsTCF3 MPQLGGGGGG GGGGSGGGGG SSAGAAGGGD DLGANDELIP FQDEGG-EQQ EPSSDSASAQ 59
HsLEF1 MPQLSGGGGG GGGDP----- ---------- ELGATDEMIP FKDEGDPQKE KIFAEISHPE 45
HsTCF1 MPQLDSGGGG AGGGD----- ---------- DLGAPDELLA FQDEGE-EGD DKSRDSAGPE 44
HsTCF4 R--DLADVKS SLVNESETNQ NSSSDSEAER RPPPRSESFR DKSRESLEEA AKRQDG-GLF 95
HsTCF3 R--DLDEVKS SLVNESENQS -SSSDSEAER RPQPVRDTFQ -KPRDYFAEV RRPQDS-AFF 114
HsLEF1 EEGDLADIKS SLVNESEIIP -ASNGHEVAR QAQTSQEPYH DKAREHPDDG -KHPDG-GLY 102
HsTCF1 R--DLAELKS SLVNESEGAA GGAGIPGVPG AGAGARGEAE ALGREHAAQR LFPDKLPEPL 102
HsTCF4 K-GPPYPGYP -FIMIPDLTS -PYLPNGSLS PT-ARTYLQM KWPLLDVQAG SLQSRQALKD 151
HsTCF3 K-GPPYPGYP -FLMIPDLSS -PYLSNGPLS PGGARTYLQM KWPLLDVP-- ---SSATVKD 166
HsLEF1 NKGPSYSSYS GYIMMPNMNN DPYMSNGSLS P--------- ---------- ---------- 133
HsTCF1 EDGLKAPECT SGMYKETVYS AFNLLMHYPP PS-------- ---------- ---------- 134
*
HsTCF4 ARSPSPAHIV SNKVPVVQHP HHVHPLTPLI TYSNEHFTPG NPPPHLPADV DPKTGIPRPP 211
HsTCF3 TRSPSPAHLS -NKVPVVQHP HHMHPLTPLI TYSNDHFSPG SPPTHLSPEI DPKTGIPRPP 225
HsLEF1 -----PIPRT SNKVPVVQPS HAVHPLTPLI TYSDEHFSPG SHPSHIPSDV NSKQGMSRHP 188
HsTCF1 ----GAGQHP QPQPPLHKAN QPPHGVPQLS LY--EHFN-S PHPTPAPADI SQKQ-VHRPL 186
HsTCF4 HPPDISPYYP LSPGTVGQIP HPLGWLVPQQ GQPVYPITT- GGFRHPYPTA LTVNASVS-- 268
HsTCF3 HPSELSPYYP LSPGAVGQIP HPLGWLVPQQ GQPMYSLPP- GGFRHPYP-A LAMNASMSSL 283
HsLEF1 PAPDIPTFYP LSPGGVGQIT PPLGW----Q GQPVYPIT-- GGFRQPYPSS LSVDTSMS-- 240
HsTCF1 QTPDLSGFYS LSLGSMGQLP HTVVWP---- SPPLYPLSPS CGYRQHFPAP TAAPGAPYP- 241
HsTCF4 ---FFPPHMV P-PHHTL-HT TGIPHPAIVT PTVKQESSQS DVGSLHSSKH QDSKKEEEKK 323
HsTCF3 VSSFFSPHMV APAHPGL-PT SGIPHPAIVS PIVKQEPAPP SLSPAVSVKS PVTVKKEEEK 342
HsLEF1 ---RFSHHMI P-GPPGP-HT TGIPHPAIVT PQVKQEHPHT DSDLMHVKPQ HEQRKEQEPK 295
HsTCF1 ---AFTHPSL MLGSGVPGHP AAIPHPAIVP PSGKQELQPF DRN---LKTQ AESKAEKEAK 295
HMG-box
HsTCF4 KPHIKKPLNA FMLYMKEMRA KVVAECTLKE SAAINQILGR RWHALSREEQ AKYYELARKE 383
HsTCF3 KPHVKKPLNA FMLYMKEMRA KVVAECTLKE SAAINQILGR KWHNLSREEQ AKYYELARKE 402
HsLEF1 RPHIKKPLNA FMLYMKEMRA NVVAECTLKE SAAINQILGR RWHALSREEQ AKYYELARKE 355
HsTCF1 KPTIKKPLNA FMLYMKEMRA KVIAECTLKE SAAINQILGR RWHALSREEQ AKYYELARKE 355
*
HsTCF4 RQLHMQLYPG WSARDNYGKK KKRKRDKQPG ETNEHSECFL NPCLSLPPIT DLSAPKKCRA 443
HsTCF3 RQLHSQLYPT WSARDNYGKK KKRKREKQLS QTQSQQ---- ---------- ---------- 438
HsLEF1 RQLHMQLYPG WSARDNYGKK KKRKREK--- ---------- ---------- ---------- 382
HsTCF1 RQLHMQLYPG WSARDNYGKK KRRSREK--- ---------- ---------- ---------- 382
HsTCF4 RFGLDQQNNW CGPCRRKKKC VRYIQGEGSC LSPPSSDGSL LDSPPPSPNL LGSPPRDAKS 503
HsTCF3 ---QVQEAEG ALASKSKKPC VQYLPPEKPC DSPASSHGSM LDSPATPSAA LASPAAPAAT 495
HsLEF1 ---LQESASG TGP----RMT AAYI------ ---------- ---------- ---------- 399
HsTCF1 -----HQEST TGG----KRN AFGTYPEKAA APAPFLPMTV L--------- ---------- 414
HsTCF4 QTEQTQPLSL SLKPDPLAHL SMMPPPPALL LAEATHKASA LCPNGALDLP PAALQPAAPS 563
HsTCF3 HSEQAQPLSL TTKPETRAQL ALHS--AAFL SAKAAASSSG QMGSQPPLLS RPLPLGSMPT 553
HsLEF1 ---------- ---------- ---------- ---------- ---------- ---------- 399
HsTCF1 ---------- ---------- ---------- ---------- ---------- ---------- 414
HsTCF4 SSIAQPS--T SWLHSHSSLA GTQPQPLSLV TKSLE 596
HsTCF3 ALLASPPSFP ATLHAHQALP VLQAQPLSLV TKSAH 588
HsLEF1 ---------- ---------- ---------- ----- 399
HsTCF1 ---------- ---------- ---------- ----- 414

Figure S3
